# Annotated checklist of Dermaptera and Zoraptera (Insecta) of Singapore, with redescriptions of several species

**DOI:** 10.3897/zookeys.1276.183701

**Published:** 2026-04-03

**Authors:** Yoshitaka Kamimura, Shen Cheah, Wendy Yanling Wang, Weng Ngai Lam

**Affiliations:** 1 Department of Biology, Keio University, Hiyoshi 4-1-1, Kohoku, Yokohama 2238521, Japan Lee Kong Chian Natural History Museum, National University of Singapore Singapore Singapore; 2 Asian School of the Environment, Nanyang Technological University, 50 Nanyang Ave, Singapore, 639798, Singapore Asian School of the Environment, Nanyang Technological University Singapore Singapore; 3 Lee Kong Chian Natural History Museum, National University of Singapore, 2 Conservatory Drive, 117377, Singapore Department of Biology, Keio University Yokohama Japan

**Keywords:** Angel insects, earwigs, endemic species, *
Gonolabis
*, new synonym, *Nannopygia ridleyi* comb. nov., urbanization

## Abstract

The earwig (Dermaptera) fauna of Singapore, previously almost entirely undocumented, is analyzed through examination of approximately 500 specimens. A critical reassessment of the limited published records is also provided. In total, 29 species identified to the species level are reported, including one Diplatyidae, two Pygidicranidae, one Apachyidae, seven Anisolabididae, two Labiduridae, nine Spongiphoridae, four Chelisochidae, and three Forficulidae. Of these, 17 species are newly recorded for Singapore: *Echinosoma roseiventre* Kamimura & Nishikawa, 2016, *Parapsalis infernalis* (Burr, 1913), *Epilandex peterseni* Ramamurthi, 1967, *Platylabia major* Dohrn, 1867, *Gonolabis minor* Borelli, 1926, *Metisolabis punctata* (Dubrony, 1879), *Nala lividipes* (Dufour, 1829), *Spirolabia pilicornis* (Motschulsky, 1863), *Paralabellula curvicauda* (Motschulsky, 1863), *Paraspania emarginata* (Srivastava, 1978), *Chaetospania javana* Borelli, 1926, *Spongovostox semiflavus* (Bormans, 1894), *Marava arachidis* (Yersin, 1860), *Proreus simulans* (Stål, 1860), *Hamaxas feae* (Bormans, 1894), *Hypurgus humeralis* (Kirby, 1891), and *Timomenus bicuspis* (Stål, 1860). Additional morphological notes are provided for selected taxa. A new synonymy is proposed, synonymizing *Gonolabis emarginata* Srivastava, 1990 with *G. sumatrana* de Bormans, 1900, resulting in *Nannopygia ridleyi* (Kirby, 1903), **comb. nov**. as the only dermapteran species currently considered endemic to Singapore. Additionally, *Spiralizoros caudelli* (Karny, 1927) is reported as the first national record of Zoraptera (angel insects). These findings are discussed in the context of Singapore’s urbanization and the biogeographic affinities of its insect fauna with those of neighboring regions.

## Introduction

The Republic of Singapore (hereafter, Singapore) is a small island nation situated at the southern tip of the Malay Peninsula, encompassing an area of ca 740 km^2^ (Department of Statistics Singapore 2026). Located near the Equator, the country was originally blanketed by tropical rainforests that supported a rich and diverse ecosystem, with tigers occupying the apex predator niche (Corlett 1992; Powell 2016). However, with its highest elevation reaching only 163 m at Bukit Timah Hill and predominantly low-lying terrain, combined with its status as the second most densely populated country in the world, the conservation of natural habitats has become an increasingly urgent concern (Castelletta et al. 2000; Brook et al. 2003; Lane et al. 2006; Theng et al. 2020; Chisholm et al. 2023; Figueroa et al. 2023). Although several nature reserves have been established in the central region of Singapore Island and ongoing surveys continue to document its wildlife, the insect fauna – particularly among smaller orders – remains poorly studied.

Dermaptera is a polyneopteran insect order with more than 2,000 described species, predominantly distributed across tropical, subtropical, and warm temperate regions (Popham 2000; Grimaldi and Engel 2005; Zhang 2013; Haas 2018; Hopkins et al. 2025). While some species are horticultural or agricultural pests, or considered as biological control agents, the majority are not directly associated with human activities (Haas 2018; Kamimura et al. 2023c). Consequently, regional faunal surveys and illustrated taxonomic references remain scarce across much of Southeast Asia.

Zoraptera (commonly known as angel insects or ground lice) is one of the smallest insect orders, with only ~ 30 extant named species, reported primarily from tropical and subtropical regions (Kočárek et al. 2020). Several phylogenetic studies have supported the hypothesis that Dermaptera and Zoraptera form a monophyletic clade within the Polyneoptera (Jarvis et al. 2005; Wipfler et al. 2020). Although four named species have been documented from Peninsular Malaysia, four from Borneo, and two from Sumatra (including the Mentawai Islands) (Kočárek et al. 2020), to the best of our knowledge, there are no published records of zorapterans from Singapore.

Drawing on literature reviews, examination of specimens from the Zoological Reference Collection (**ZRC**) housed at the Lee Kong Chian Natural History Museum (**LKCNHM**, National University of Singapore), and the authors’ own field surveys, we present a comprehensive list of Dermaptera and Zoraptera species recorded from Singapore. Redescriptions are provided for several species, with a proposed new synonymy. Revised synonymic lists are also included for all species identified to the species level.

## Materials and methods

Dermapteran and zorapteran specimens from the ZRC were examined, excluding a subset of several material stored in ethanol. Additional specimens were collected from leaf litter in forested areas of the Central Catchment Nature Reserve and Pulau Ubin using Winkler extractors during ecological studies conducted between 2021 and 2024. Opportunistic bycatches from other insect sampling methods including flight intercept traps (FIT), pitfall traps, and others were also examined. An intensive field survey was conducted by all authors from 7–11 March 2024, with all collected specimens subsequently deposited in the ZRC. We also critically reviewed published records of Dermaptera and Zoraptera from Singapore, along with the known distributions of species encountered during the field survey. Monographs and compilations by Sakai (1985, 1987, 1990, 1991, 1992, 1993, 1994, 1995a, 1995b, 1995c, 1995d, 1996), Steinmann (1986, 1989a, 1989b, 1990, 1993), and Srivastava (1988, 2003b, 2013) were extensively used as primary taxonomic references.

For selected representative specimens, genitalia were dissected under a stereomicroscope (M80; Leica Microsystems, Germany), mounted in CV Ultra mounting medium (Leica Biosystems, Germany) between two coverslips, and pinned alongside the respective specimen. Genital structures were observed using a light microscope (BX50; Olympus, Tokyo, Japan) equipped with an Olympus DP27 CCD camera. Focused image segments were combined using Combine ZP Image Stacking Software (Hadley 2010). Composite images of external morphology were obtained using the microscope mode and focus-stacking sub-mode of a Tough TG-5 digital camera (Olympus) or a Leica M205A microscope equipped with a Flexacam C1 imaging system (Leica Camera AG, Wetzlar, Germany).

In the following section, records based on specimens deposited in the ZRC and NTU (= currently housed at Nanyang Technological University) are presented with unique catalog numbers in brackets, respectively. Specimens examined in this study from regions other than Singapore are presented throughout the main text, with their depository information and catalog codes.

For cosmopolitan species, certain dermapteran taxa have extensive numbers of synonyms. Therefore, references are provided only for the original publications in which each scientific name combination first appeared in synonym lists. Due to the presence of multiple errors in synonym lists published in earlier monographs, a revised and verified synonymy (with the type locality for each scientific name) is presented here, accompanied by complete bibliographic citations.

Regarding previous distribution records, Malaysian data are separated into Peninsular Malaysia and Borneo. Records from Indonesia are categorized by major islands or island groups (e.g., Java, Sumatra, Sulawesi). For Borneo (Kalimantan) and New Guinea, where national boundaries are often ambiguous in historical records, data are presented by island rather than by country.

Suprageneric classification follows Kamimura (2025), while generic classification is based on Srivastava (1988, 1996, 1999, 2003b, 2013), unless otherwise specified. Terminology for female and male genital structures follows Klass (2003) and Kamimura (2025), respectively.

### Depository acronyms and other abbreviations

**CAS** California Academy of Sciences, USA

**FMNH** Field Museum of Natural History, Chicago, USA

**NHM** The Natural History Museum, London, UK

**NHMD** Natural History Museum of Denmark, University of Copenhagen, Denmark

**NTU** Collection at Nanyang Technological University

**ZRC** Zoological Reference Collection, Lee Kong Chian Natural History Museum, National University of Singapore, Singapore

**FIT** Flight intercept trap

**gl9** gonoplac (= coxal lobe) IX

**gp8** gonapophysis VIII

**leg** legit: name of the person who collected this specimen

**sp** spermatheca.

## Results

### Order DERMAPTERA de Geer, 1773

**Suborder Neodermaptera Engel, 2003**

**Infraorder Protodermaptera Zacher, 1910**

**Superfamily Pygidicranoidea Verhoeff, 1902a**

**Family Diplatyidae Verhoeff, 1902a**

**Subfamily Diplatyinae Verhoeff, 1902a**

***Nannopygia* Dohrn, 1863a (reinstated as a senior synonym of *Schizodiplatys* Steinmann, 1974: Srivastava 1993)**

#### Records in Singapore

##### Nannopygia ridleyi

Taxon classificationAnimaliaDermapteraDiplatyidae

(Kirby, 1903) comb. nov.

01920D43-FF8C-5AE2-94DD-5164FA72C61E

Figs 1–7

Diplatys ridleyi Kirby, 1903: 61 (Singapore).

###### Literature.

Kirby (1903: 61): Singapore (as *Diplatys rideleyi*)

###### Specimens examined.

**ZRC** • 1 ♀; Bukit Timah Forest; 16 Feb. 1983; D.H. Murphy leg.; Tree bole with termite plastering; ZRC_DER_0000156. • 1 ♂; same data as for ZRC_DER_0000156; ZRC_DER_0000157.

###### Description of male

(ZRC_DER_0000156). Length of body (without forceps): 10.4 mm. Length of forceps: 1.8 mm. Head width: 1.4 mm. Pronotum width: 1.0 mm. Pronotum length: 1.1 mm.

Color (Figs 2, 3): generally dark brown; mouth parts, antennae (2^nd^ segment onward), legs pale brown, except dark brownish band on mid femur and base of tibia. Metazona of pronotum, scutellum, and hindwings (except fustis) whitish. Abdomen and forceps reddish brown. Head (Fig. 2): coriaceous with sparse setae, approx. as long as broad, widest in eye region; frons tumid and occiput depressed; transverse suture obsolete, median suture distinct; hind margin widely emarginated in middle; post-ocular carina visible but not prominent, almost straight, running from middle of the internal margin of eyes to the hind margin of the head. Antennae partly broken (only 10 segments remaining in both right and left antenna); first segment stout, expanded apically, length ~ 1/2 of the distance between the antennal bases; second segment almost quadrate, small; third segment 1.5× longer than broad, fourth almost quadrate, shorter than third; fifth segment nearly equal to third, remaining segments gradually lengthening and narrowing distally. Eyes prominent, slightly longer than postocular length. Pronotum (Fig. 2): smooth, approx. as long as broad; anterior and posterior margins widely and gently rounded; sides almost straight, narrowing towards posterior; fore and hind angles rounded; median sulcus distinct; prozona raised and metazona depressed. Tegmina (Fig. 2): well-developed, ~ 2.5× longer than the pronotum, finely punctulate; humeral angle weak; posterior margins broadly rounded. Hindwings well-developed; length of exposed part almost same as pronotum. Legs long, slender; hind tarsi with first segment ~ 2× as long as third, second segment ~ 1/2 as long as third, claw with arolium. Abdomen (Fig. 2): long, cylindrical, shiny but sparsely punctulate, segments seventh onward gradually expanded. Penultimate sternite (= sternite IX) (Fig. 3): almost quadrate, parallel sided, posterior margin almost truncate with rounded angles and feebly emarginate at middle. Ultimate tergite (Fig. 2): approx. as long as broad, gradually sloping backwards; lateral margins almost straight in anterior 2/3 then oblique; hind margin with a pair of swellings at the bases of forceps. Forceps symmetrical, widely separated and strongly curved inward at middle. Genitalia (Figs 4–6): virga with thin, bifurcated tubes, ~ 2× the length of penis lobe, ellipsoid vesicle at base followed by long common duct, bifurcated part ~ 1/5 of entire virgal length, with inner branch conspicuously thicker and longer than outer branch; penis lobe also encloses spindle-shaped sclerite on the outer side; inner side with a smaller rod-shaped sclerite; parameres (Fig. 6) deeply cleft; outer parameral lobe curving inward, acuminate; inner parameral lobe 2× broader and 1.5× longer than outer lobe, spatula-shaped but with acute apex pointing inward.

**Figures 1–10.**
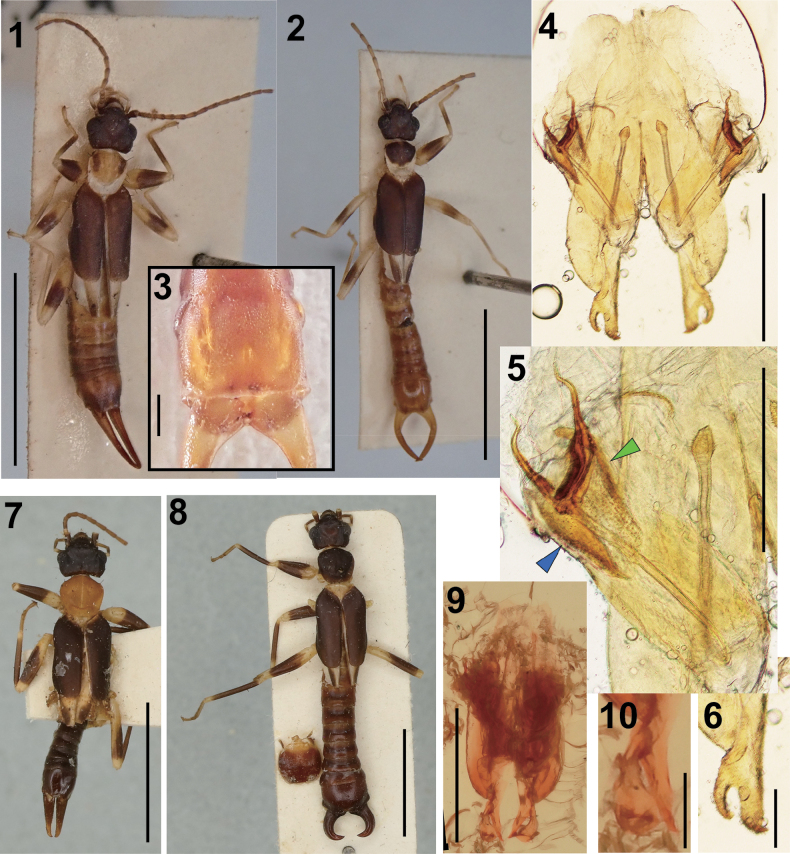
**1–7***Nannopygia ridleyi* (Kirby, 1903), comb. nov. **1**. Female dorsal habitus ( ZRC_DER_0000156); **2**. Male dorsal habitus (ZRC_DER_0000157); **3**. Male penultimate sternite; **4**. Male genitalia; **5**. Left virga; **6**. Left paramere; **7**. Female habitus (holotype: NHMUK 013323041); **8–10**. *Nannopygia malayanus* (Hincks, 1957) (holotype: male: NHMUK 013323046): **8**. Dorsal habitus and penultimate sternite **9**. Genitalia; **10**. Left paramere. The blue and green arrowheads in **5** indicate the spindle- and lod-shaped accessory sclerites in a penis lobe, respectively. Scale bars: 5 mm (**1, 2, 7, 8**); 1 mm (**4, 9**); 0.5 mm (**3, 5**); 0.2 mm (**6, 10**).

###### Redescription of female

(ZRC_DER_0000157)

Length of body (without forceps): 8.1 mm. Length of forceps: 1.7 mm. Head width: 1.3 mm. Pronotum width: 1.0 mm. Pronotum length: 1.1 mm.

Coloration similar to males but prozona of pronotum paler (Fig. 1). Eyes smaller, approx. as long as postocular length. Forceps (Fig. 1) almost straight, slender, tapering.

###### Distribution.

Singapore.

###### Remarks.

The external morphology of the adult female specimen from the ZRC closely matches that of the holotype female of *Diplatys ridleyi* described by Kirby (1903; NHMUK 013323041, Natural History Museum (2025): Fig. 7). Although the paler regions of the holotype appear slightly more yellow, such variation in coloration is common within this group and may reflect the number of days elapsed since emergence and/or preservation environments. Accordingly, we identify both the male and female specimens in the ZRC as belonging to *Diplatys ridleyi*. Since the original description, based on a holotype collected from an unspecified locality in Singapore, no subsequent records of *D. ridleyi* have been reported (see Hincks 1955), and the male has remained undescribed. The male specimen described herein exhibits deeply cleft (i.e., bilobed) parameres, a diagnostic feature of the genus *Nannopygia* Dohrn, 1863a (= *Schizodiplatys* Steinmann, 1974). We thus propose *Nannopygia ridleyi* (Kirby, 1903), comb. nov.

Hincks (1957) described *Nannopygia malayanus* (as *Diplatys malayanus*) based on a single male specimen collected at Kuala Lumpur, Peninsular Malaysia. He also treated his *Diplatys* sp. (Hincks 1955) from Mt. Ophir (= Gunung Ledang), Johor State, Peninsular Malaysia, ca 180 km northwest from the center of Singapore Island as a synonym of *N. malayanus* (Hincks 1957). These two species are very similar, including in the structure of the male genitalia, and are clearly closely related. However, our comparison with the holotype of *N. malayanus* (NHMUK 013323046: Figs 8–10) reveals the following differences: (1) outer lobes of the parameres wider than the inner lobes in *N. malayanus* (vs inner lobes wider in the specimen from Singapore); (2) the tips of outer lobes rounded, forming a paddle shape in *N. malayanus* (vs both inner and outer lobes curve inwardly and acuminate, forming a hook at their apex); (3) inner and outer branches of the bifurcated part of virgae almost same length in *N. malayanus* (vs longer inner branches); and (4) metazona of pronotum and tarsi much more strongly marked in black in *N. malayanus*. While this final difference could be attributable to individual variation, the structural differences in (1) to (3) are significant enough to conclude that the two names should not be considered synonyms.

Similarly, the external morphology and genitalia of the Singaporean male and female specimens examined in the present study closely resemble those of *N. karnyi* (Borelli, 1926) known from Indonesia (Mentawai Islands) (Borelli 1926a; Hincks 1955). However, the male of *N. karnyi* differs in the following respects: (1) the apex of the outer branch of parameres is thick and almost truncate (vs tapering and distinctly pointed apically in the specimen from Singapore); (2) within each penis lobe, three spine-like (?) structures overlapping the respective virga are present (vs such structures absent); (3) the inner and outer branches of the bifurcated virgae are approximately equal in length (vs the inner branch distinctly longer); and (4) the posterior margin of the penultimate sternite is broadly rounded, with a large oval depression anterior to it (vs nearly truncate with a faint, oval marking). The last character, however, may be attributable to the fact that the specimen from Singapore represents the macrolabic form—within Diplatyidae, intraspecific variation between the macro-, meso-, and microlabic forms has been reported in some species (Hincks 1955; Sakai 1985). The shape of the paramere is also similar to those of *N. angustatus* (Burr, 1910) from Bangladesh (Burr 1910b), but this species is characterized by the unique shape of the penultimate sternite with the caudal margin protruding medially, forming a subtruncate lobe (Hincks 1955). *Nannopygia* appears to have diversified into numerous closely related species in Southeast Asia forming the *nigriceps* group (Hincks 1955). The present identification is based primarily on the concordance of female external morphology (except for the coloration of the pronotum) among specimens collected from the same locality, rather than on a positive determination derived from detailed examination of male morphology. Further studies on intraspecific variation based on a larger number of specimens, as well as comparative DNA analyses, are desirable.

### Family Pygidicranidae Verhoeff, 1902a

**Subfamily Pygidicraninae Verhoeff, 1902a**

***Cranopygia* Burr, 1908c**

#### Cranopygia

Taxon classificationAnimaliaDermapteraPygidicranidae

sp.

A330FCE4-9C45-5D81-B523-FA7948F733A4

##### Specimens examined.

**NTU** • 1 ♀; Upper Seletar Reservoir Park; 19 Mar. 2024; S. Yap leg.; FIT; EB_FIT285_24.

##### Remarks.

Only one adult female sample, degraded and lacking the mid abdomen, was collected using an FIT.

### Subfamily Echinosomatinae Burr, 1910b

***Echinosoma* Audinet-Serville, 1839**

#### Echinosoma roseiventre

Taxon classificationAnimaliaDermapteraPygidicranidae

Kamimura & Nishikawa, 2016

2089D347-4FDA-5F96-A948-22B87B3AF90F

Figs 11–15

Echinosoma roseiventre Kamimura et al., 2016b: 53, figs 1a, 2–9 (Peninsular Malaysia, Penang Island).
Echinosoma sp.: Kamimura et al. 2016a: 240 (Peninsular Malaysia, Penang Island).

##### Specimens examined.

**ZRC** • 1 ♂; MacRitchie Nature Park; 7 Mar. 2024; Y. Kamimura leg.; Hand collection (day); ZRC_ENT00064395. • 1 ♀ (winged form); same data as for ZRC_ENT00064395; ZRC_ENT00064396. • 1 ♀; same data as for ZRC_ENT00064395; ZRC_ENT00064397. • 1 nymph; Thomson Nature Park; 12 Mar. 2024; Y. Kamimura leg.; Hand collection (day); ZRC_ENT00064428.

##### Description of females

(ZRC_ENT00064396, ZRC_ENT00064397)

Length of body (without forceps): 7.3–7.6 mm. Length of forceps: 0.6–0.7 mm. Head width: 1.3 mm. Pronotum width: 1.2 mm. Pronotum length: 0.95 mm.

**Figures 11–15.**
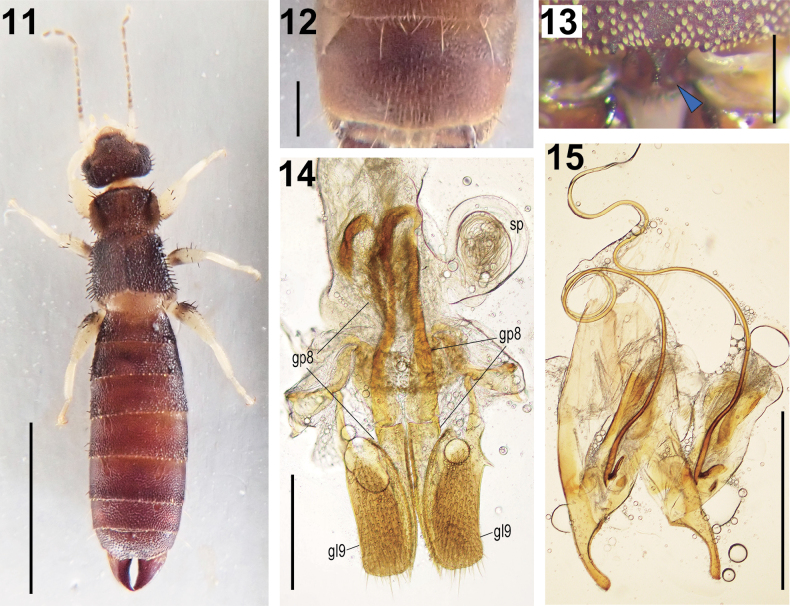
*Echinosoma roseiventre* Kamimura & Nishikawa, 2016. **11–14**. Female (brachypterous form: ZRC_ENT00064397): **11**. Dorsal habitus; **12**. Penultimate sternite; **13**. Pygidium; **14**. Genitalia; **15**. Male (brachyoterous form: ZRC_ENT00064395) genitalia. The blue arrowhead in **13** indicates the projection on female pygidium. Abbreviations: gl9, gonoplac (= coxal lobe) IX; gp8, gonapophysis VIII; sp, spermatheca. Scale bars: 3 mm (**11**); 0.5 mm (**12–15**).

Color: General body color (Fig. 11) dull smoky black but abdomen, especially caudal part, pygidium, and forceps reddish brown or rosy. Mouth parts brownish. Antennae dark brown except for first three segments dirty white. Legs dirty white but femora with a broad fuscous band near the base. Caudal margin of tegmina with distinct, narrow, whitish band. First abdominal segment whitish. Body sparsely covered with obtuse bristles. Head (Fig. 11): approx. as long as broad; frons convex; transverse and median suture indistinct; caudal margin feebly emarginated in middle. Antennae: 17 segments; first expanded apically, ~ 1/2 long as the distance between antennal bases; second short, quadrate; third long, 2× as long as broad; fourth and fifth short, as long as broad; sixth and beyond gradually longer and narrowing basally rendering some segments subpyriform. Eyes long but shorter than post-ocular length. Post-ocular margin with a row of five or six long bristles. Pronotum (Fig. 11): broader than long; surface rough; lateral sides rounded; frontal and caudal angles weakly and strongly rounded, respectively; caudal margin convex with distinct emargination in middle; prozona distinctively raised; median sulcus weak but visible; row of long bristles on frontal and lateral margins. Tegmina (Fig. 11): longer than pronotum; surface rough; humeral angle weak and anal angle shortly rounded off to show a small, triangular scutellum; caudal margin obliquely truncate, outer and caudal margins with long bristles. In the winged female, hindwing scales protruding from tegmina, ~ 1.5× longer than tegmina. Legs (Fig. 11): stout; femora not compressed, ecarinate; arolium small; hind tarsi with first segment longer than third. Abdomen (Fig. 11): fusiform, tergites with scattered granules and very short obtuse bristles with whitish apex; first two tergites and lateral sides of third tergites onward with long bristles near caudal margins. Penultimate sternite (Fig. 12): transverse, rectangular with caudal margin almost straight. Ultimate tergite (Fig. 11): transverse, with small projections above the base of forceps; caudal margin feebly emarginated at middle. Pygidium (Fig. 13): short, with a small triangular projection dorsally, but hidden when observed from the dorsal side. Forceps thicker than those of males, almost triangular, bases in close proximity, tips curving inward, creating a spindle-shaped space between them. Genitalia (Fig. 14) with well-developed gonapophyses (particularly gonoplac IX and gonapophysis VIII), as is usual in this genus. Spermatheca (Fig. 14): elongate, tubular, without a pigmented capsule at the distal end.

##### Distribution.

Malaysia (Penang Island), Singapore (new record).

##### Remarks.

The external and genital features of a male (ZRC_ENT00064395: Fig. 15) match those of *E. roseiventre* described by Kamimura et al. (2016b), for which only two brachypterous males have been reported to date from Penang Island, Malaysia. Two females, one of which with fully developed wings, were collected along with the male during a field survey. The description of the female is provided for the first time. A nymph, with well-developed wing buds, was also tentatively identified as this species (ZRC_ENT00064428).

This species closely resembles *E. andamanense* Srivastava, 1988 (originally described as *E. andamanensis*: see Nishikawa 2023), which was described from India (Kamimura et al. 2016b). The females of the species described herein also exhibit similar external morphology; however, the pygidium of *E. andamanense* is notably larger and longer, making it visible from the dorsal side (Srivastava 1988).

### Subfamily Prolabiscinae Bey-Bienko, 1959b

***Parapsalis* Borelli, 1921**

#### Parapsalis infernalis

Taxon classificationAnimaliaDermapteraPygidicranidae

(Burr, 1913)

CB4FB2A6-BB62-54EA-955D-535BFF12611F

Chaetospania infernalis Burr, 1913b: 67, fig. 3 (Formosa [= Taiwan]).
Chaetospania ? sp. Burr, 1913a: 142 (Arunachal Pradesh, India).
Parapsalis laevis Borelli, 1921: 3, fig. 1 (Sandakan, Borneo).
Protolabis aroliata Bey-Bienko, 1959a: 598, figs 10, 14 (Yunnan, China).
Prolabisca aroliata : Bey-Bienko 1959b: 943.
Anisolabis infernalis : Sakai 1970a: 69.
Anisolabis laevis : Sakai 1970a: 69.
Prolabisca infernalis : Nishikawa 1976: 44.
Parapsalis infernalis : Steinmann 1989b: 188.

##### Specimens examined.

**ZRC** • 1 ♂; Thomson Nature Park; 8 Mar. 2024; Y. Kamimura leg.; Hand collection (day); ZRC_ENT00064409. • 1 ♂; same data as for ZRC_ENT00064409; ZRC_ENT00064410. • 1 ♂; same data as for ZRC_ENT00064409; ZRC_ENT00064411. • 1 ♀; same data as for ZRC_ENT00064409; ZRC_ENT00064412. • 1 ♀; same data as for ZRC_ENT00064409; ZRC_ENT00064413. • 1 ♀; same data as for ZRC_ENT00064409; ZRC_ENT00064414. • 1 ♀; same data as for ZRC_ENT00064409; ZRC_ENT00064415. • 1 ♂; same collection data as for ZRC_ENT00064409; 12 Mar. 2024; ZRC_ENT00064434. • 1 ♂; same data as for ZRC_ ZRC_ENT00064434; ZRC_ENT00064435. • 1 ♂; same data as for ZRC_ ZRC_ENT00064434; ZRC_ENT00064436. • 1 ♀; same data as for ZRC_ ZRC_ENT00064434; ZRC_ENT00064437. • 1 ♀; same data as for ZRC_ ZRC_ENT00064434; ZRC_ENT00064438. • 1 nymph; same data as for ZRC_ ZRC_ENT00064434; ZRC_ENT00064439.

##### Distribution.

Japan (Ryukyu Islands), Taiwan, China, Bhutan, India, Vietnam, Thailand, Peninsular Malaysia, Borneo, Sumatra, Singapore (new record).

##### Remarks.

This unique species constitutes the monotypic subfamily Prolabiscinae (Kamimura 2025).

### Superfamily Apachyoidea Verhoeff, 1902a

**Family Apachyidae Verhoeff, 1902a**

***Apachyus* Audinet-Serville, 1831**

#### Apachyus chartaceus

Taxon classificationAnimaliaDermapteraApachyidae

(de Haan, 1842)

601117E4-4200-5762-A61B-BA46FB091C6E

Forficula chartacea de Haan, 1842: 239, pl. 23 fig. 7 (Sumatra).
Apachya chartacea : Dohrn 1863a: 43.
Apachys chartacea : Dubrony 1879a: 349.
Apachyus chartaceus : de Bormans and Krauss 1900: 13.

##### Specimens examined.

**ZRC** • 1 ♀; Thomson Nature Park, Lorong Pelita; 8 Mar. 2024; Y. Kamimura leg.; Waste woodland forest, under thick bark of large fallen tree (*Terminalia catappa*), hand collection (day); ZRC_ENT00064426. • 1 ♀; Thomson Nature Park, Lorong Pelita; 12 Mar. 2024; Y. Kamimura leg.; Waste woodland forest, under thick bark of large fallen tree (*Terminalia catappa*), hand collection (day); ZRC_ENT00076889 (formerly in a YK personal collection as YK_Sin2024_1). • 1 ♂; same data as for preceding; ZRC_ENT00076890 (formerly YK_Sin2024_2). • 1 ♀; same collection data as for preceding; ZRC_ENT00076783 (formerly YK_Sin2024_3). • 1 nymph; same collection data as for preceding; ZRC_ENT00076784 (formerly YK_Sin2024_4).

##### Distribution.

Java, Borneo, Sumatra, Peninsular Malaysia, Singapore (new record).

##### Remarks.

Kamimura (2025) reported two samples (one of each sex) collected during the intensive field survey conducted in 2024. The author proposed placing Apachyidae (*Apachyus* and *Dendroiketes* Burr, 1909), Gonolabiidae Popham & Brindle, 1966b (*Gonolabina* Verhoeff, 1902b), and Allostethidae Verhoeff, 1904 (*Allostethus* Verhoeff, 1904, *Allostethella* Zacher, 1910, and *Gonolabidura* Zacher, 1910) in Infraorder Protodermaptera, based mainly on their neck and genital structures.

#### Apachyus

Taxon classificationAnimaliaDermapteraPygidicranidae

sp.

EDEB53B7-EA1F-59F9-833E-AA8B3DA6A05B

##### Specimens examined.

**ZRC** • 2 nymphs; Venus Drive; 4 Sep. 2014; M.S. Foo leg.; Tree bark; ZRC_ENT00007592.

##### Remarks.

Possibly nymphs of *A. chartaceus*, but further studies required.

### Infraorder Epidermaptera Engel, 2003

**Parvorder Mesodermaptera Steinmann, 1986**

**Superfamily Anisolabidoidea Verhoeff, 1902a**

**Family Anisolabididae Verhoeff, 1902a**

**Subfamily Platylabiinae Burr, 1911b**

***Platylabia* Dohrn, 1867**

#### Platylabia major

Taxon classificationAnimaliaDermapteraAnisolabididae

Dohrn, 1867

05A5F2B5-FEA1-5F44-B0A2-84494BA16B6F

Platylabia major Dohrn, 1867: 347 (Sulawesi).
Labidophora major : Scudder 1876c: 297.
Platylabia sparattoides de Bormans, 1900: 459 (Penang Island and Sumatra).
Palex sparattoides : Burr 1910b: 68, fig. 77.

##### Specimens examined.

**ZRC** • 1 nymph; Bukit Timah Forest; 3 Nov. 1974; D.H. Murphy leg.; Under bark; ZRC_DER_0000107. • 1 ♂; Bukit Timah Forest; 3 Nov. 1974; D.H. Murphy leg.; Under bark; ZRC_DER_0000121. • 1 ♂; same data as for ZRC_DER_0000121; ZRC_DER_0000122. • 1 ♂; same data as for ZRC_DER_0000121; ZRC_DER_0000123. • 1 ♂; same data as for ZRC_DER_0000121; ZRC_DER_0000124. • 1 ♀; same data as for ZRC_DER_0000121; ZRC_DER_0000160. • 1 ♀; same data as for ZRC_DER_0000121; ZRC_DER_0000161. • 1 nymph; same data as for ZRC_DER_0000121; ZRC_DER_0000162. • 1 ♀; same data as for ZRC_DER_0000121; ZRC_DER_0000163. • 1 ♀; Bukit Timah Forest; 3 Dec. 1974; D.H. Murphy leg.; ZRC_DER_0000164. • 1 ♀; Bukit Timah Forest. *S. slope*; 3 Dec. 1974; D.H. Murphy leg.; ZRC_DER_0000165. • 1 ♀; Windsor Nature Park; 7 Mar. 2024; Y. Kamimura leg.; Hand collection (day); ZRC_ENT00064394.

##### Distribution.

Widely distributed in the Indomalayan region, including China, Vietnam, Myanmar, Thailand, Peninsular Malaysia, Borneo, Sumatra, Java, Sulawesi, and Singapore (new record).

### Subfamily Anisolabidinae Verhoeff, 1902a

***Epilandex* Hebard, 1927**

#### Epilandex peterseni

Taxon classificationAnimaliaDermapteraAnisolabididae

Ramamurthi, 1967

B90C9D69-DC74-5FEB-9B46-6A383F54F863

Figs 16–21

Epilandex peterseni Ramamurthi, 1967: 234, figs 6–8 (Tawi Tawi, Philippines)

##### Specimens examined.

**ZRC** • 1 ♀; Lower Kent Ridge Rd; 4 Dec. 1991; Samantha Lee leg.; ZRC_6_16342. • 1 ♀; Kent Ridge; 5 Jan. 1992; Jean W.H. Yong leg.; ZRC_DER_0000057. • 1 ♂; Kent Ridge; 3 Nov. 1991; Jean W.H. Yong leg.; ZRC_DER_0000060. **NTU** • 1 ♂; Central catchment nature reserve; 2023; S. Yap leg.; Flight intercept trap; FIT183_23_1.

##### Distribution.

Philippines (Tawi Tawi, Palawan, Mindanao), Bismarck Islands (New Britain, New Ireland), Borneo (Brunei), Singapore (new record).

##### Remarks.

**Figures 16–21.**
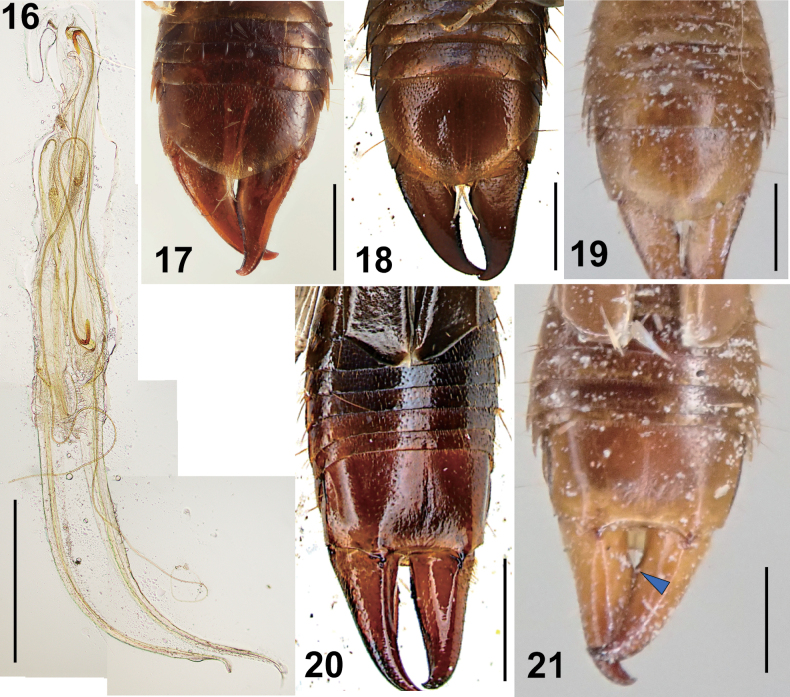
Male *Epilandex peterseni* Ramamurthi, 1967. **16**. Genitalia (NTU AA_FIT183_23); **17**. Post abdomen (ventral view: zmuc00028762); **18**. Post abdomen (ventral view: NTU AA_FIT183_23); **19**. Post abdomen (ventral view: ZRC_DER_0000058); **20**. Post abdomen (dorsal view: NTU AA_FIT183_23); **21**. Post abdomen (dorsal view: ZRC_DER_0000058). The blue arrowhead in **21** indicates concavements on the inner margin of forceps. Scale bars: 1 mm.

According to Kočárek (2021) and Srivastava (1999), the genus *Epilandex* includes nine species distributed across Indomalayan, Australian, and Oceanian regions, and five species from South America. Although previous researchers described the penultimate sternite of male *E. peterseni* as bearing a rudimentary carina at the posterior apex (Ramamurthi 1967; Srivastava 2003a; Kočarek 2021), examination of the holotype (male: zmuc00028762 [NHMD]) reveals the absence of a carina. Instead, the posterior margin of the penultimate sternite is broadly rounded, with only a very weak median protrusion (Fig. 17). In light of this, the morphological characteristics of the specimens from Singapore (Figs 16, 18–21) are fully consistent with the description of *E. peterseni* provided by Ramamurthi (1967) and subsequent authors (Srivastava 2003a; Kočarek 2021). Although Ramamurthi (1967) described only broken genitalia for the male holotype, the genitalia of the Singapore specimen (NTU FIT183_23_1) correspond well with the original description, including the presence of very long parameres and virgae, the base of the latter being strongly recurved (Fig. 16). While the inner margin of the male forceps has been described as entire in the literature, some Singaporean specimens exhibit a weakly concave base (Fig. 21). Similarly, the development of a median longitudinal carina on each side of abdominal tergites VI–IX shows considerable variation among the examined males (Figs 17–21).

### *Euborellia* Burr, 1910c

#### Euborellia annulata

Taxon classificationAnimaliaDermapteraAnisolabididae

(Fabricius, 1793)

2A3A36EC-C8AA-5789-B588-40FCBA7FA32D

Forficula annulata Fabricius, 1793: 4 (Americae meridionalis [= West Indies]).
Forcinella stali Dohrn, 1864a: 286 (Java).
Anisolabis stali : Scudder 1876c: 305.
Anisolabis minuta Caudell, 1907: 168 (Puerto Rico).
Borellia stali : Burr 1910b: 88.
Euborellia stali : Burr 1911b: 31.
Euborellia minuta : Rehn and Hebard 1917: 639.
Euborellia annulata : Brindle 1981: 14.

##### Literature.

Burr (1902: 479) as *Anisolabis stali*: “*A. Ståli* Dohrn. – Singapore (Biró)”

##### Specimens examined.

**ZRC** • 1 ♂; Linden Drive; 8 Feb. 1975; D.H. Murphy leg.; ZRC_DER_0000125. • 1 ♂; same data as for ZRC_DER_0000125; ZRC_DER_0000126. • 1 ♂; University ground; May 1965; P.H.*L. leg*.; ZRC_DER_0000127. • 1 ♀; Linden Drive; 7 Dec. 1981; D.H. Murphy leg.; Under dog dung; ZRC_DER_0000146. • 1 ♂; same data as for ZRC_DER_0000146; ZRC_DER_0000150.

##### Distribution.

Widely distributed in tropical and subtropical regions of the world.

##### Remarks.

A circumtropical cosmopolitan species, usually found in lowlands including seashores (Kamimura et al. 2023a).

### *Gonolabis* Burr, 1900b

#### Gonolabis electa

Taxon classificationAnimaliaDermapteraAnisolabididae

Burr, 1910

1D3C1132-DC8B-5196-8383-608E20C60019

Gonolabis electa Burr, 1910b: 79, fig. 21 (Sri Lanka).
Gonolabis calas Fernando, 1961: 42, pl. 3, figs 6–8 (Sri Lanka).

##### Literature.

Borelli (1932a: 82): “Botanic Gardens, Novembre 1926. 1♂. (C. Dover).”

##### Specimens examined.

**ZRC** • 2 ♂♂, 2 ♀♀; Bukit Timah Hindhede Drive; NA Dec. 1989; H.K. Lua leg.; ZRC_DER_0000001. • 1 ♀; Lower Peirce Reservoir, Forest A; 11 Aug. 1990; ZRC_DER_0000035. • 1 ♀; Fort Canning Park; 24 Jan. 1990; W.H. Ho leg.; Inside shell of *Dyakia* sp.; ZRC_DER_0000053. • 1 ♂; Along View Rd; 29 Sep. 2012; M.S. Foo leg.; ZRC_DER_0000063. • 1 ♂; Bukit Timah Hindhede Drive; 27 Aug. 1989; H.K. Lua leg.; ZRC_DER_0000075. • 1 ♀; same data as for ZRC_DER_0000075; ZRC_DER_0000078. • 1 ♂; Bukit Timah Forest, Taban Valley; 6 Jun. 1976; D.H. Murphy leg.; Attracted to very old fish; ZRC_DER_0000128. **NTU** • 1 ♀; Dairy Farm nature park; 15 Oct. 2021; W.N. Lam, G.K. Png, S.K.B. Tan leg.; Winkler extraction of leaf litter; BT2F_21_1. • 1 ♂; Dairy Farm nature park; 18 Apr. 2023; W.N. Lam, G.K. Png, J. L. Loo leg.; Winkler extraction of leaf litter; BT2F_23_1. • 1 ♀; same data as for BT2F_21_1; BT2U_21_1. • 1 ♂; same data as for BT2F_23_1; BT2U_23_1. • 1 ♂; same data as for BT2F_23_1; BT2U_23_2. • 1 ♀; same data as for BT2F_23_1; BT2U_23_3. • 1 ♀; same data as for BT2F_23_1; BT2U_23_4. • 1 ♀; same data as for BT2F_23_1; BT2U_23_5. • 1 ♂; Central catchment nature reserve; 27 Oct. 2021; W.N. Lam, G.K. Png, S.K.B. Tan leg.; Winkler extraction of leaf litter; MAC1F_21_1. • 1 ♀; same data as for MAC1F_21_1; MAC1F_21_2. • 1 ♀; Central catchment nature reserve; 10 Apr. 2023; W.N. Lam, G.K. Png, J. L. Loo leg.; Winkler extraction of leaf litter; MAC1F_23_1. • 1 ♀; same data as for MAC1F_23_1; MAC1F_23_2. • 1 ♀; same data as for MAC1F_21_1; MAC1U_21_1. • 1 ♂; same data as for MAC1F_23_1; MAC1U_23_1. • 1 ♂; same data as for MAC1F_23_1; MAC1U_23_2. • 1 ♀; same data as for MAC1F_23_1; MAC1U_23_3. • 1 ♀; same data as for MAC1F_23_1; MAC1U_23_4. • 1 ♀; same data as for MAC1F_23_1; MAC1U_23_5. • 1 ♀; same data as for MAC1F_23_1; MAC1U_23_6. • 1 ♀; same data as for MAC1F_23_1; MAC1U_23_7. • 1 ♂; same data as for MAC1F_23_1; MAC2U_23_2. • 1 ♀; same collection data as for MAC1F_23_1; 16 May 2023; PEI2F_23_2. • 1 ♂; same data as for PEI2F_23_2; PEI2F_23_3. • 1 ♀; same data as for PEI2F_23_2; PEI2F_23_4. • 1 ♂; same data as for PEI2F_23_2; PEI2U_23_2. • 1 ♂; same data as for PEI2F_23_2; PEI2U_23_3. • 1 ♂; same data as for PEI2F_23_2; PEI2U_23_4. • 1 ♀; same data as for PEI2F_23_2; PEI2U_23_5. • 1 ♂; same data as for PEI2F_23_2; PEI2U_23_6. • 1 ♀; same data as for PEI2F_23_2; PEI2U_23_7. • 1 ♀; same data as for PEI2F_23_2; PEI2U_23_8. • 1 ♂; same collection data as for MAC1F_23_1; 23 May 2023; PEI5F_23_2. • 1 ♀; same data as for PEI5F_23_2; PEI5F_23_3. • 1 ♂; same data as for PEI5F_23_2; PEI5F_23_4. • 1 ♂; same data as for PEI5F_23_2; PEI5F_23_5. • 1 ♂; same data as for PEI5F_23_2; PEI5F_23_6. • 1 ♀; same data as for PEI5F_23_2; PEI5F_23_7. • 1 ♀; same data as for PEI5F_23_2; PEI5F_23_8. • 1 ♂; same collection data as for MAC1F_21_1; 6 Oct. 2021; PEI5U_21_1. • 1 ♂; same collection data as for MAC1F_21_1; 8 Oct. 2021; PEI7U_21_1. • 1 ♀; same data as for PEI7U_21_1; PEI7U_21_2. • 1 ♂; Pulau Ubin; 10 May 2023; W.N. Lam, G.K. Png, J. L. Loo leg.; Winkler extraction of leaf litter; UBI10F_23_1. • 1 ♂; same collection data as for UBI10F_23_1; 8 May 2023; UBI1U_23_1. • 1 ♀; Pulau Ubin; 11 Nov. 2021; W.N. Lam, G.K. Png, S.K.B. Tan leg.; Winkler extraction of leaf litter; UBI2F_21_1. • 1 ♂; same data as for UBI2F_21_1; UBI2F_21_2. • 1 ♂; same collection data as for UBI10F_23_1; 9 May 2023; UBI2F_23_1. • 1 ♀; same data as for UBI2F_21_1; UBI2U_21_1. • 1 ♀; same data as for UBI2F_23_1; UBI2U_23_1. • 4 ♂♂; same data as for UBI1U_23_1; UBI4U_23_1-4. • 1 ♀; same data as for UBI1U_23_1; UBI4U_23_5. • 1 ♂; same data as for UBI1U_23_1; UBI5U_23_1. • 2 ♂♂; same collection data as for UBI2F_21_1; 17 Nov. 2021; UBI7F_21_2-3. • 1 ♂; same data as for UBI10F_23_1; UBI7F_23_1. • 1 ♀; same data as for UBI10F_23_1; UBI7F_23_2. • 1 ♀; same data as for UBI7F_21_2-3; UBI7U_21_1. • 1 ♀; same data as for UBI7F_21_2-3; UBI7U_21_2. • 1 ♀; same data as for UBI10F_23_1; UBI7U_23_1. • 1 ♀; same data as for UBI10F_23_1; UBI7U_23_2. • 1 ♂; same collection data as for UBI10F_23_1; 3 May 2023; UBI9U_23_7.

##### Distribution.

Sri Lanka, Vietnam, Peninsular Malaysia, Java, Sumatra, Lesser Sunda Islands, Philippines, India (Kerala: Emiliyamma 2017), Seychelles, Singapore. Also adventive along the West African coast (Brindle 1978), including Mauritius (Srivastava 1984).

##### Remarks.

This species is ubiquitous in the leaf litter layers of secondary forests across Singapore, and also often occurs in urban parks and gardens.

#### Gonolabis minor

Taxon classificationAnimaliaDermapteraAnisolabididae

Borelli, 1926

3970914C-9123-5566-8D0A-5D05858E8C44

Figs 22–25

Gonolabis minor Borelli, 1926c: 253 (Malabar, Java).

##### Specimens examined.

**NTU** • 1 ♀; Central catchment nature reserve; 10 Apr. 2023; W.N. Lam, G.K. Png, J. L. Loo leg.; Winkler extraction of leaf litter; MAC2U_23_1. • 1 ♂; same data as for MAC2U_23_1; MAC3U_23_2. • 1 ♂; Central catchment nature reserve; 21 Oct. 2021; W.N. Lam, G.K. Png, S.K.B. Tan leg.; Winkler extraction of leaf litter; MAC6U_21_1.

##### Distribution.

Java, Sunda Islands, Philippines (Luzon), Singapore (new record).

##### Remarks.

**Figures 22–25.**
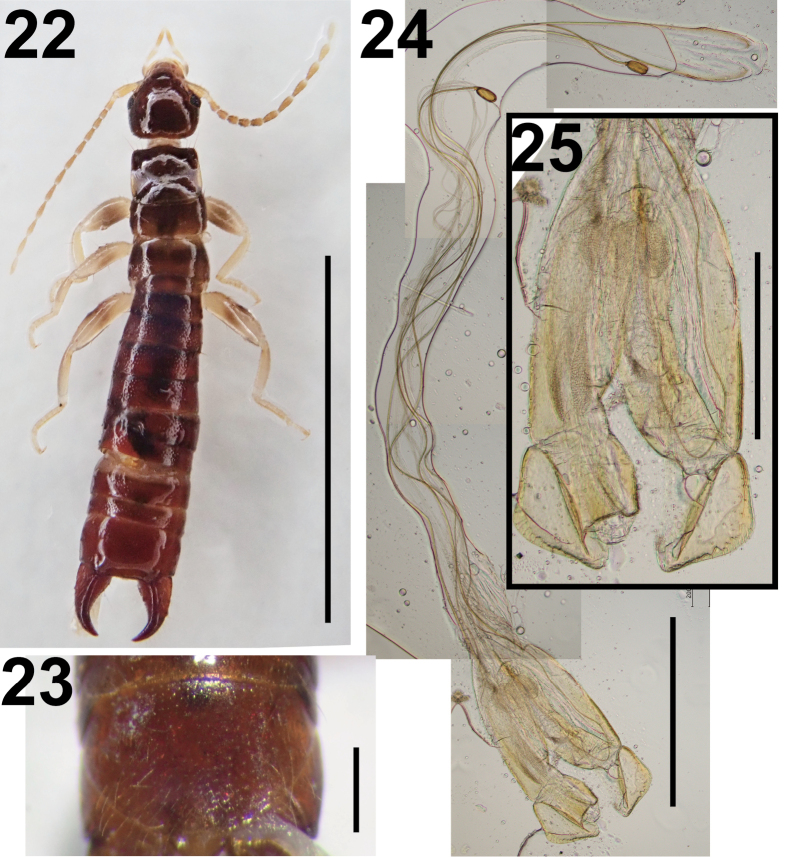
*Gonolabis minor* Borelli, 1926 (male: NTU MAC6U_21_1). **22**. Dorsal habitus; **23**. Penultimate sternite; **24**. Genitalia; **25**. Distal part of genitalia. Scale bars: 5 mm (**22**); 1 mm (**24**); 0.5 mm (**23, 25**).

The external morphology of two adult males from Singapore corresponds closely to that of *Gonolabis minor* as described by Borelli (1926c; original description), Srivastava (1983), and Sakai (1987). Diagnostic features include the smaller body size relative to *G. electa* (Fig. 22) and a penultimate sternite with a broadly rounded posterior margin (Fig. 23). Borelli’s type series of this species consists of only two syntype specimens, a single male and a single female in Museo Regionale di Scienze Naturali, Torino, Italy (Srivastava 1983). The original description by Borelli (1926c) did not include illustrations of the male genitalia, which had already been removed and were missing from the male syntype. These structures are illustrated here for the first time (Figs 24, 25). The penis lobes lack conspicuous internal structures such as accessory sclerites or denticulated pads, and instead contain only a thin virga. The outer margin of the parameres, unlike the rounded form seen in *G. sumatrana*, consists of nearly straight lines (Fig. 25), consistent with Borelli (1926c).

Adults of this species can be readily distinguished from *G. sumatrana* (= *G. emarginata*) by their markedly smaller body size, and from *G. electa* by the presence of *Euborellia*-like, triangular parameres in males. However, females of similar size within *Gonolabis* are morphologically indistinct, complicating species-level identification. Preliminary DNA analysis confirms that one female specimen (NTU MAC2U_23_1) from Singapore belongs to *G. minor*. This species appears to be restricted to primary and late-successional secondary forests, in contrast to its congeners *G. electa* and *G. sumatrana*, which are commonly found in young secondary forests and abandoned plantation habitats.

#### Gonolabis sumatrana

Taxon classificationAnimaliaDermapteraAnisolabididae

de Bormans, 1900

625BA4EA-F6D3-52C4-A7FF-14EBDCB3C927

Figs 26–33

Gonolabis sumatrana de Bormans, 1900: 452 (Sumatra).
Gonolabis emarginata Srivastava, 1990: 27, figs 12–17 (Bukit Timah, Singapore). syn. nov.

##### Literature.

Burr (1902: 480) “*G. sumatrana* Borm. (fig. 2. ♂). – Singapore (Biró).”

##### Specimens examined.

**ZRC** • 2 ♂♂, 2 ♀♀, 1 nymph; Bukit Timah Hindhede Drive; Dec. 1989; H.K. Lua leg.; ZRC_DER_0000003. • 1 ♀; Lower Peirce Reservoir; 21 Jul. 1990; C.M. Yang leg.; ZRC_DER_0000006. • 1 ♂, 1 ♀; Pulau Tekong; 27 Nov. 2001; C.M. Yang, C.M.*S. leg*.; Under honey comb, Drain; ZRC_DER_0000024. • 1 ♂; Botanic Gardens; Jun. 1965; P.H.*L. leg*.; ZRC_DER_0000129. **NTU** • 1 ♀; Central catchment nature reserve; 10 Apr. 2023; W.N. Lam, G.K. Png, J. L. Loo leg.; Winkler extraction of leaf litter; MAC2F_23_1. • 1 ♂; same data as for MAC2F_23_1; MAC2F_23_2. • 1 ♀; same data as for MAC2F_23_1; MAC3U_23_1. • 1 ♂; same collection data as for MAC2F_23_1; 14 Apr. 2023; MAC6(re)F_23_1. • 1 ♀; Central catchment nature reserve; 21 Oct. 2021; W.N. Lam, G.K. Png, S.K.B. Tan leg.; Winkler extraction of leaf litter; MAC6F_21_1. • 1 ♂; same collection data as for MAC6F_21_1; 4 Oct. 2021; PEI1F_21_1. • 1 ♂; same collection data as for MAC2F_23_1; 23 May 2023; PEI6F_23_1. • 1 ♀; Pulau Ubin; 10 May 2023; W.N. Lam, G.K. Png, J. L. Loo leg.; Winkler extraction of leaf litter; UBI10U_23_1. • 1 ♂; same collection data as for UBI10U_23_1; 8 May 2023; UBI1F_23_1. • 1 ♀; same collection data as for UBI10U_23_1; 12 May 2023; UBI3U_23_2. • 1 ♂; Pulau Ubin; 17 Nov. 2021; W.N. Lam, G.K. Png, S.K.B. Tan leg.; Winkler extraction of leaf litter; UBI7F_21_1. • 1 ♀; same collection data as for UBI10U_23_1; 3 May 2023; UBI9U_23_1.

##### Distribution.

Peninsular Malaysia, Borneo, Sumatra, Java, Sulawesi, Singapore.

##### Remarks.

**Figures 26–34.**
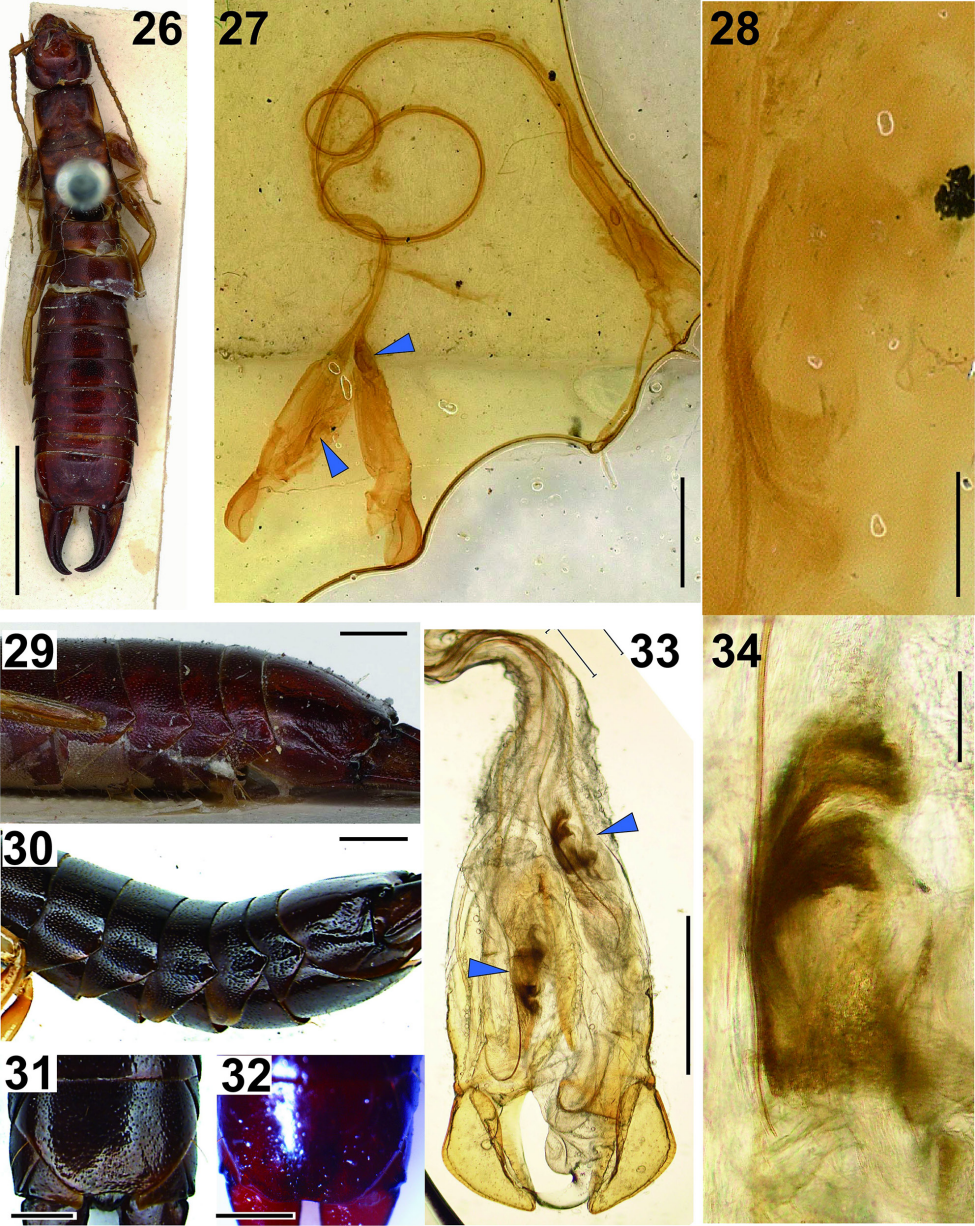
Male *Gonolabis sumatrana* de Bormans, 1900. **26–29**. Holotype of *Gonolabis emarginata*Srivastava 1990 syn. nov. (CASTYPE16526): **26**. Dorsal habitus; **27**. Genitalia; **28**. Accessory sclerite of penis lobe; **29**. Post abdomen (left lateral view). **30, 31, 33, 34**. Male (Singapore: NTU PEI6F23(1)): **30**. Post abdomen (left lateral view); **31**. Penultimate sternite; **33**. Genitalia (distal part); **34**. Accessory sclerite of penis lobe; **32**. Male (ZRC_DER_0000003) penultimate sternite. The blue arrowheads in **27** and **33** indicate the accessory sclerite in each penis lobes. Scale bars: 5 mm (**26**); 1 mm (**27, 29–33**); 0.1 mm (**28, 34**).

Srivastava (1990) described *Gonolabis emarginata* based on a single adult male specimen collected at Bukit Timah, Singapore (Figs 26–29). He noted that the species closely resembles *G. sumatrana*, but distinguished it by a larger body size, a slightly emarginate posterior margin of the penultimate sternite (vs broadly rounded in *G. sumatrana*), and parameres with a concave internal margin and rounded external angle (vs straight and obtuse). However, examination of multiple *G. sumatrana* specimens from Singapore revealed that the posterior margin of the penultimate sternite is nearly truncate at the middle in smaller males (Fig. 32) but tends to be concave in larger males, with the variation appearing continuous (Fig. 31). Additionally, the paramere morphology of the *G. emarginata* holotype (CASTYPE16526 [CAS]) falls within the observed range of individual variation in *G. sumatrana* (Figs 27, 33; see also Sakai 1987). Both taxa consistently exhibit a comma-shaped sclerite within each penis lobe along with a virga (Figs 28, 34; Zacher 1911). Although Srivastava (1990) noted similarities between *G. emarginata* and *G. oblita* Burr, 1910a, such accessory structures have not been reported for *G. oblita* (Burr 1915; Brindle 1980).

Preliminary DNA analysis further supports the synonymy, revealing minimal genetic variation among *G. sumatrana* specimens from Singapore, including individuals with *G. emarginata*-like traits. Based on these findings, we conclude that *G. emarginata* represents a large-bodied variant of *G. sumatrana*, and we propose its treatment as a junior synonym of the latter. It is also noteworthy that *G. sumatrana* exhibits a sharp posterior projection on the lateral sides of abdominal segments VI through IX, with the degree of projection varying individually and typically more pronounced in larger males (Figs 29, 30). According to Burr (1915), *G. oblita* lacks such structures.

Previous records of *G. sumatrana* from Borneo and Peninsular Malaysia (Borelli 1932a, b) are based solely on female specimens, which are difficult to identify to species level and warrant further examination. However, a male specimen in the ZRC likely collected in Pahang, Peninsular Malaysia (ZRC_DER_0000008), may provide additional insights.

#### Anisolabidinae

Taxon classificationAnimaliaDermapteraAnisolabididae

sp.

6C0A7E02-FD8E-5A9A-8739-47A1BC0A8870

##### Specimens examined.

**NTU** • 1 ♀; Central catchment nature reserve; 30 May 2023; W.N. Lam, G.K. Png, J. L. Loo leg.; Winkler extraction of leaf litter; PEI8F_23_1. • 1 ♂; same data as for PEI8F_23_1; PEI8F_23_2. • 1 ♀; same data as for PEI8F_23_1; PEI8U_23_1.

##### Remarks.

Preliminary molecular analyses imply the presence of an additional species of *Gonolabis*-like anisolabidids, the identification of which will require further material for confirmation.

#### Anisolabidinae spp.

Taxon classificationAnimaliaDermapteraAnisolabididae

7A484214-94D1-597F-90BB-F1B3FFC80B7E

##### Specimens examined.

**ZRC** • 1 nymph; Lower Peirce Reservoir; 21 Jul. 1990; C.M. Yang leg.; ZRC_DER_0000007. • 1 nymph; MacRitchie Reservoir; 5 Oct. 2012; X.R. Ong leg.; Cattle dung pitfall trap; ZRC_DER_0000065. • 1 nymph; same collection data as for ZRC_DER_0000065; 9 Sep. 2011; ZRC_DER_0000067. **NTU** • 1 nymph; Bukit Timah nature reserve; 17 Apr. 2023; W.N. Lam, G.K. Png, J. L. Loo leg.; Winkler extraction of leaf litter; BT1F_23_1. • 1 nymph; Dairy Farm nature park; 15 Oct. 2021; W.N. Lam, G.K. Png, S.K.B. Tan leg.; Winkler extraction of leaf litter; BT2F_21_2. • 5 nymphs; same collection data as for BT1F_23_1; 18 Apr. 2023; BT2F_23_2-6. • 1 nymph; same data as for BT2F_21_2; BT2U_21_2. • 10 nymphs; same data as for BT2F_21_2; BT2U_21_3-12. • 1 nymph; same data as for BT2F_23_2-6; BT2U_23_6. • 1 nymph; same data as for BT2F_23_2-6; BT2U_23_7. • 1 nymph; Central catchment nature reserve; 27 Oct. 2021; W.N. Lam, G.K. Png, S.K.B. Tan leg.; Winkler extraction of leaf litter; MAC1F_21_3. • 7 nymphs; Central catchment nature reserve; 10 Apr. 2023; W.N. Lam, G.K. Png, J. L. Loo leg.; Winkler extraction of leaf litter; MAC1F_23_3-9. • 1 nymph; same data as for MAC1F_21_3; MAC1U_21_2. • 1 nymph; same data as for MAC1F_21_3; MAC1U_21_3. • 7 nymphs; same data as for MAC1F_23_3-9; MAC1U_23_8-14. • 1 nymph; same data as for MAC1F_23_3-9; MAC2U_23_3. • 1 nymph; same data as for MAC1F_23_3-9; MAC3F_23_1. • 1 nymph; same data as for MAC1F_23_3-9; MAC3F_23_2. • 1 nymph; same data as for MAC1F_23_3-9; MAC3U_23_3. • 1 nymph; same data as for MAC1F_23_3-9; MAC3U_23_4. • 1 nymph; same data as for MAC1F_23_3-9; MAC3U_23_5. • 1 nymph; same collection data as for MAC1F_21_3; 12 Oct. 2021; MAC4F_21_1. • 1 nymph; same collection data as for MAC1F_23_3-9; 12 Apr. 2023; MAC4F_23_1. • 1 nymph; same collection data as for MAC1F_21_3; 13 Oct. 2021; MAC5F_21_1. • 1 nymph; same data as for MAC5F_21_1; MAC5F_21_2. • 1 nymph; same data as for MAC4F_23_1; MAC5F_23_1. • 1 nymph; same data as for MAC4F_23_1; MAC5F_23_2. • 1 nymph; same data as for MAC4F_23_1; MAC5F_23_3. • 1 nymph; same data as for MAC5F_21_1; MAC5U_21_1. • 1 nymph; same collection data as for MAC1F_23_3-9; 14 Apr. 2023; MAC6(re)F_23_2. • 1 nymph; same collection data as for MAC1F_21_3; 21 Oct. 2021; MAC6F_21_2. • 1 nymph; same data as for MAC6(re)F_23_2; MAC6F-HC_23_5. • 1 nymph; same data as for MAC6(re)F_23_2; MAC6F-HC_23_6. • 1 nymph; same data as for MAC6(re)F_23_2; MAC6F-HC_23_7. • 1 nymph; same data as for MAC6F_21_2; MAC6U_21_2. • 1 nymph; same data as for MAC6F_21_2; MAC6U_21_3. • 1 nymph; same data as for MAC6F_21_2; MAC6U_21_4. • 1 nymph; same collection data as for MAC1F_23_3-9; 13 Apr. 2023; MAC7U_23_1. • 1 nymph; same data as for MAC6F_21_2; MAC8F_21_1. • 1 nymph; same collection data as for MAC1F_21_3; 4 Oct. 2021; PEI1F_21_2. • 1 nymph; same data as for PEI1F_21_2; PEI1U_21_1. • 1 nymph; same collection data as for MAC1F_23_3-9; 16 May 2023; PEI2F_23_5. • 1 nymph; same data as for PEI2F_23_5; PEI2F_23_6. • 1 nymph; same data as for PEI2F_23_5; PEI2F_23_7. • 1 nymph; same data as for PEI2F_23_5; PEI2F_23_8. • 1 nymph; same data as for PEI2F_23_5; PEI2F_23_9. • 1 nymph; same data as for PEI2F_23_5; PEI2U_23_10. • 1 nymph; same data as for PEI2F_23_5; PEI2U_23_11. • 1 nymph; same data as for PEI2F_23_5; PEI2U_23_9. • 1 nymph; same collection data as for MAC1F_21_3; 5 Oct. 2021; PEI3F_21_1. • 4 nymphs; same data as for PEI2F_23_5; PEI3F_23_1-4. • 1 nymph; same data as for PEI2F_23_5; PEI3U_23_1. • 1 nymph; same data as for PEI2F_23_5; PEI3U_23_2. • 1 nymph; same collection data as for MAC1F_23_3-9; 2023; PEI3U_23_3. • 1 nymph; same collection data as for MAC1F_23_3-9; 23 May 2023; PEI4F_23_1. • 7 nymphs; same data as for PEI4F_23_1; PEI4U_23_1-7. • 1 nymph; same collection data as for MAC1F_21_3; 6 Oct. 2021; PEI5F_21_1. • 1 nymph; same data as for PEI5F_21_1; PEI5F_21_2. • 1 nymph; same data as for PEI5F_21_1; PEI5F_21_3. • 1 nymph; same data as for PEI4F_23_1; PEI5F_23_1. • 10 nymphs; same data as for PEI4F_23_1; PEI5F_23_9-18. • 1 nymph; same data as for PEI4F_23_1; PEI5SEQAU_23_1. • 1 nymph; same data as for PEI4F_23_1; PEI5SEQAU_23_2. • 1 nymph; same data as for PEI4F_23_1; PEI5SEQAU_23_3. • 1 nymph; same data as for PEI4F_23_1; PEI5SEQAU_23_4. • 1 nymph; same data as for PEI4F_23_1; PEI5SEQBU_23_1. • 1 nymph; same data as for PEI4F_23_1; PEI5SEQBU_23_2. • 1 nymph; same data as for PEI4F_23_1; PEI5SEQBU_23_3. • 1 nymph; same data as for PEI5F_21_1; PEI5U_21_2. • 1 nymph; same data as for PEI5F_21_1; PEI6F_21_1. • 1 nymph; same data as for PEI4F_23_1; PEI6F_23_2. • 1 nymph; same data as for PEI4F_23_1; PEI6U_23_1. • 1 nymph; same collection data as for MAC1F_21_3; 8 Oct. 2021; PEI7F_21_1. • 1 nymph; same data as for MAC1F_23_3-9; 11 Apr. 2023; PEI7F_23_1. • 1 nymph; same data as for PEI7F_23_1; PEI7F_23_2. • 1 nymph; same data as for PEI7F_23_1; PEI7F_23_3. • 1 nymph; same data as for PEI7F_23_1; PEI7F_23_4. • 1 nymph; same data as for PEI7F_23_1; PEI7F_23_5. • 1 nymph; same data as for PEI7F_23_1; PEI7U_23_1. • 1 nymph; same data as for PEI7F_23_1; PEI7U_23_2. • 1 nymph; same collection data as for MAC1F_21_3; 11 Oct. 2021; PEI8F_21_1. • 1 nymph; same data as for MAC1F_23_3-9; 30 May 2023; PEI8F_23_3. • 1 nymph; same data as for PEI8F_23_3; PEI8F_23_4. • 1 nymph; same data as for PEI8F_23_3; PEI8F_23_5. • 1 nymph; same data as for PEI8F_23_3; PEI8U_23_2. • 1 nymph; same data as for PEI8F_23_3; PEI9U_23_1. • 1 nymph; same data as for PEI8F_23_3; PEI9U_23_2. • 1 nymph; Pulau Ubin; 18 Nov. 2021; W.N. Lam, G.K. Png, S.K.B. Tan leg.; Winkler extraction of leaf litter; UBI10F_21_1. • 1 nymph; same data as for UBI10F_21_1; UBI10F_21_3. • 16 nymphs; Pulau Ubin; 10 May 2023; W.N. Lam, G.K. Png, J. L. Loo leg.; Winkler extraction of leaf litter; UBI10F_23_2-17. • 1 nymph; same data as for UBI10F_23_2-17; UBI10U_23_2. • 1 nymph; same collection data as for UBI10F_21_1; 11 Nov. 2021; UBI1F_21_1. • 1 nymph; same collection data as for UBI10F_23_2-17; 8 May 2023; UBI1U_23_2. • 4 nymphs; same data as for UBI1U_23_2; UBI1U_23_3-6. • 2 nymphs; same data as for UBI1F_21_1; UBI2F_21_3-4. • 8 nymphs; same collection data as for UBI10F_23_2-17; 9 May 2023; UBI2F_23_2-9. • 1 nymph; same data as for UBI1F_21_1; UBI2U_21_2. • 1 nymph; same data as for UBI2F_23_2-9; UBI2U_23_2. • 1 nymph; same data as for UBI2F_23_2-9; UBI2U_23_3. • 1 nymph; same collection data as for UBI10F_23_2-17; 12 May 2023; UBI3AF_23_1. • 1 nymph; same data as for UBI3AF_23_1; UBI3BF_23_1. • 1 nymph; same data as for UBI3AF_23_1; UBI3BF_23_2. • 1 nymph; same data as for UBI3AF_23_1; UBI3BF_23_3. • 1 nymph; same collection data as for UBI10F_21_1; 15 Nov. 2021; UBI3U_21_1. • 1 nymph; same data as for UBI3U_21_1; UBI3U_21_2. • 1 nymph; same data as for UBI3U_21_1; UBI3U_21_3. • 1 nymph; same collection data as for UBI10F_23_2-17; 12 May 2023; UBI3U_23_3. • 1 nymph; same data as for UBI3U_23_3; UBI3U_23_4. • 1 nymph; same data as for UBI3U_23_3; UBI3U_23_5. • 1 nymph; same collection data as for UBI10F_23_2-17; 8 May 2023; UBI4F_23_1. • 1 nymph; same data as for UBI4F_23_1; UBI4F_23_2. • 1 nymph; same data as for UBI4F_23_1; UBI4F_23_3. • 6 nymphs; same data as for UBI4F_23_1; UBI4U_23_6-11. • 1 nymph; same collection data as for UBI10F_21_1; 17 Nov. 2021; UBI5U_21_1. • 1 nymph; same data as for UBI4F_23_1; UBI5U_23_2. • 1 nymph; same data as for UBI5U_21_1; UBI6F_21_1. • 7 nymphs; same collection data as for UBI10F_23_2-17; 3 May 2023; UBI6F_23_1-7. • 1 nymph; same data as for UBI5U_21_1; UBI6U_21_1. • 1 nymph; same data as for UBI6F_23_1-7; UBI6U_23_1. • 1 nymph; same data as for UBI6F_23_1-7; UBI6U_23_2. • 4 nymphs; same data as for UBI5U_21_1; UBI7F_21_4-7. • 5 nymphs; same collection data as for UBI10F_23_2-17; 10 May 2023; UBI7F_23_3-7. • 14 nymphs; same data as for UBI7F_23_3-7; UBI7U_23_3-16. • 1 nymph; same data as for UBI6F_23_1-7; UBI9F_23_1. • 1 nymph; same data as for UBI6F_23_1-7; UBI9F_23_2. • 1 nymph; same data as for UBI6F_23_1-7; UBI9F_23_3. • 1 nymph; same data as for UBI6F_23_1-7; UBI9F_23_4. • 1 nymph; same data as for UBI6F_23_1-7; UBI9F_23_5. • 1 nymph; same collection data as for UBI10F_21_1; 18 Nov. 2021; UBI9U_21_1. • 1 nymph; same data as for UBI6F_23_1-7; UBI9U_23_2. • 1 nymph; same data as for UBI6F_23_1-7; UBI9U_23_3. • 1 nymph; same data as for UBI6F_23_1-7; UBI9U_23_4. • 1 nymph; same data as for UBI6F_23_1-7; UBI9U_23_5. • 1 nymph; same data as for UBI6F_23_1-7; UBI9U_23_6. • 1 nymph; Central catchment nature reserve; 2024; S. Yap leg.; Hand collected, from deadwood; USP11.01_1. • 1 nymph; same data as for USP11.01_1; USP8.01_1.

##### Remarks.

Due to the inherent difficulty in identifying juvenile specimens of this subfamily to the species or genus level, they are treated as unidentified.

### Subfamily Brachylabidinae Burr, 1908b

***Metisolabis* Burr, 1910b**

#### Metisolabis punctata

Taxon classificationAnimaliaDermapteraAnisolabididae

(Dubrony, 1879)

007C8D24-3348-5ED5-8FFA-3FC0EABE9FF6

Brachylabis punctata Dubrony, 1879a: 357, fig. (Java).
Leptisolabis punctata : Burr, 1911b: 43.
Isolabis punctata : Popham and Brindle 1966a: 245.
Ctenisolabis punctata : Steinmann, 1978: 217.
Isolabis (Ctenisolabis) punctata : Sakai, 1982: 24.

##### Specimens examined.

**NTU** • 1 ♂; Central catchment nature reserve; 16 May 2023; W.N. Lam, G.K. Png, J.L. Loo leg.; Winkler extraction of leaf litter; PEI2U_23_1.

##### Distribution.

North Australia, New Guinea, Sumatra, Java, Peninsular Malaysia, Singapore (new record).

##### Remarks.

The identification of the samples is only tentative, because of the many similar species in the genus that are inadequately described.

### Superfamily Labiduroidea Verhoeff, 1902a

**Family Labiduridae Verhoeff, 1902a**

**Subfamily Labidurinae Verhoeff, 1902a**

***Labidura* Leach, 1815**

#### Labidura riparia

Taxon classificationAnimaliaDermapteraLabiduridae

(Pallas, 1773)

44B92F1D-3D10-57B8-980E-804BDE51AA2B

Forficula riparia Pallas, 1773: 727 (Irtysh river, Western Siberia).
Forficula pallipes Fabricius, 1775: 270 (Insulis promontrii viridis [= Cape Verde Islands]).
Forficula bilineata Herbst, 1786: 183, pl. 49, fig. 1 (type locality: unknown).
Forficula gigantea Fabricius, 1787: 224 (Europa auftraliori [= Southern Europe]).
Forficula maxima Villiers, 1789: 427 (Occitania [= southern France]).
Forficula bidens Olivier, 1791: 466 (Jamaica).
Forficula crenata Olivier, 1791: 467 (southern Africa).
Forficula flavipes Fabricius, 1793: 2 (Guinea).
Forficula erythrocephala Fabricius, 1793: 4 (Americae meridionalis [= West Indies]).
Forficula rufescens Palisot de Beauvois, 1805: 35, Orthoptères pl. 1, fig. 2 (“A Oware” of Africa).
Labidura gigantea : Leach 1815: 118.
Psalis morbida Audinet-Serville, 1831: 35 (type locality: unknown).
Forficesila bivittata Klug, in Burmeister, 1838: 751 (Santo Domingo, Puerto Rico, and Colombia).
Forficesila gigantea : Burmeister 1838: 751.
Forficesila suturalis Burmeister, 1838: 752 (Colombia).
Forficula marginella Costa, 1839: 50 (Mount Vesuvius, Italy).
Forficesila terminalis Audinet-Serville, 1839: 25 (Île de France).
Forficesila icterica Audinet-Serville, 1839: 25 (Pondicherry, India).
Forficula (Forficesila) gigantea var. japonica de Haan, 1842: 240 (Japan).
Forficula bicolor Fischer, 1846: 42 (Tauride [= Crimean Peninsula]).
Forficesila riparia : Fischer 1846: 46.
Forficula fischeri Motschulsky in Fischer, 1846: 354 (Tauride [= Crimean Peninsula]).
Forficula (Forficesila) affinis Guérin-Méneville, 1856: in Sagra 1856: 137, pl. 12, fig. 2 (Cuba).
Forficula amurensis Motschulsky, 1859: 499 (Amur).
Labidura riparia : Dohrn 1863b: 313.
Labidura servillei Dohrn, 1863b: 316 (Madras, Eastern India [= Chennai, India]).
Labidura auditor Scudder, 1876a: 252 (Natal [= KwaZulu-Natal Province, South Africa]).
Labidura livida Dubrony, 1879b: 93 (Santa Catarina, Brazil)
Labidura riparia var. *inermis*Brunner von Wattenwyl 1882: 5 (Europe).
Labidura glanulosa Kirby, 1891: 511 (Philippines).
Labidura pluvialis Kirby, 1891: 512 (Raine Island [=Raine Island, Queensland, Australia]).
Labidura (?) *clarki* Kirby, 1891: 512 (Rio Janeiro[= Rio de Janeiro, Brazil]).
Labidura distincta Rodzianko, 1897: 153 (Transcaucasia, Dshewat Province, Baku [= Baku, Azerbaijan]).
Labidura riparia var. mixta Bolívar 1897: 117 (Cádiz, Spain).
Labidura riparia livida : de Bormans and Krauss 1900: 35.
Labidura riparia japonica : de Bormans and Krauss 1900: 35.
Labidura riparia erythrocephala : de Bormans and Krauss 1900: 35.
Labidura riparia pluvialis : de Bormans and Krauss 1900: 35.
Labidura servillei icterica : de Bormans and Krauss 1900: 36.
Apterygida huseinae Rehn, 1901: 273 (“Gallaland”, northeast Africa).
Labidura truncata Kirby, 1903: 67 (Australia).
Labidura icterica var. japonica : Kirby 1904: 11.
Tomopygia sinensis Burr, 1904: 288 (Pekin, China [= Beijing, China]).
Labidura dubronyi Borg, 1904: 565 (Cameroon).
Labidura karschi Borg, 1904: 566, Pl. 26, fig. 1 (Cameroon).
Labidura mongolica Rehn, 1906: 503, fig. 2 (Peking, Chi-li, China [= Beijing, China]).
Labidura riparia var. dumonti Azam 1906: 80 (Elisa-Vethpol, Caucase [= Caucasus]).
Labidura riparia herculeana Semenov, 1908: 171 (Deserta Kirgisorum in finibus prov. Uralensis [= Kyrgyz Republic]).
Labidura japonica : Burr, 1911b: 37.
Labidura bidens : Hebard 1917b: 411.
Labidura leucotarsata Mjöberg, 1913: 27 (Perth, southwestern Australia).
Labidura confusa Capra, 1929: 157, fig. 20c (Giarabub, Egypt [= Jaghbub, Libya]).
Labidura riparia truncata : Hebard 1933: 147.

##### Literature.

Neo and Su (2024): “At least 15 examples, each about 2 cm in length, consisting of adult and immature individuals” at “Pasir Ris Park, Sungei Tampines; 31 December 2023; around 2223 hrs”

##### Specimens examined.

**ZRC** • 1 ♀; Sembawang; 1 Sep. 2000; L.M. Kok leg.; Seashore; ZRC_DER_0000059. • 1 nymph; NUH garden; 8 Nov. 2012; P.O. Lau leg.; ZRC_DER_0000068. • 1 nymph; Pulau Ubin; 8 Nov. 1988; ZRC_DER_0000069.

##### Distribution.

Cosmopolitan.

##### Remarks.

A cosmopolitan species widely recorded from subboreal to tropical regions. However, a recent study implies the occurrence of several cryptic species (Kamimura et al. 2023b), warranting further studies on the Singaporean population of this species.

### Subfamily Nalinae Steinmann, 1975

***Nala* Zacher, 1910**

#### Nala lividipes

Taxon classificationAnimaliaDermapteraLabiduridae

(Dufour, 1928)

3A9C41D9-7119-52B6-B245-B86A9AA10F64

Forficula pallipes Dufour, 1820: 316, pl. 96, fig. 7 (Espagne [= Spain])
*“Forficule de Dufour”* [= Forficula dufouri] Desmarest, 1820: pl. 1 fig. 7
Forficula lividipes Dufour, 1828: 340. [replacement name for Forficula pallipes Dufour, 1820: 316 preoccupied by Forficula pallipes Fabricius, 1775: 270] (Spain).
Forficesila meridionalis Audinet-Serville, 1839: 26 (Espagne [= Spain]).
Forficesila castanea Audinet-Serville, 1839: 26 (type locality: unknown) .
Forficesila vicina Lucas, 1846: 5, pl. 1, fig. 2 (Algiers and El Kala, Algeria).
Labidura pallipes : Dohrn, 1863b: 317.
Labidura vicina : Dohrn 1863b: 318.
Labidura duforii : Scudder 1876c: 322.
Echinosoma (?) *obscurum* Kirby, 1900 in Distant (1900): 12, pl. 1 fig. 2 (Pretoria, Trasvaal [= Pretoria, South Africa]).
Labidura inconspicua Kirby, 1900 in Distant (1900): 13, pl. 1 fig. 1 (Pretoria, Trasvaal [= Pretoria, South Africa]).
Labidura lividipes : de Bormans and Krauss 1900: 36.
Paralabidura lividipes : Burr 1910a: 185.
Nala lividipes : Zacher 1910: 30.
Labidura australica Mjöberg, 1913: 27 (West Kimberley, northwest Australia).

##### Specimens examined.

**ZRC** • 1 ♀; NUH garden; 10 Sep. 2012; P.O. Lau leg.; Hand caught; ZRC_DER_0000055. • 1 ♂; Upper Jurong Rd, NTI Campus; 2 Nov. 1980; Y.H. Koo leg.; ZRC_DER_0000089. • 1 ♀; Bukit Timah; 7 Oct. 1980; L.G. Yeoh leg.; ZRC_DER_0000094.

##### Distribution.

Widely distributed throughout Tropical Asia, Southern Europe, Africa, and Australia. New record to Singapore.

### Parvorder Eudermaptera Verhoeff, 1902a

**Superfamily Forficuloidea Latreille, 1810**

**Family Spongiphoridae Verhoeff, 1902a**

**Subfamily Nesogastrinae Verhoeff, 1902a**

***Nesogaster* Verhoeff, 1902a**

#### Nesogaster amoenus

Taxon classificationAnimaliaDermapteraSpongiphoridae

(Stål, 1855)

5CE020E0-C1EC-591C-BA8E-06AD69CAD4E0

Forficula amoena Stål, 1855: 350 (Java).
Labia amoena : Dohrn 1864b: 425.
Nesogastrella ruficeps Verhoeff, 1902a: 192 (Borneo).
Labia pulchripes de Bormans in Burr, 1903: 236 (Australie boréale [= northern Australia]).
Nesogaster amoenus : Burr 1908a: 46.
Nesogaster pulchripes : Burr 1908a: 46.
Nesogaster gonopygius Borelli, 1926a: 389, fig. (Siberut, Mentawai Islands, west Sumatra, Indonesia).

##### Literature.

Kirby (1904: 26) as *Labia amoena*.

##### Specimens examined.

**ZRC** • 1 ♂; Venus Drive; 3 Nov. 2012; Phira unadirekkul leg.; On fungus on log; ZRC_DER_0000064. • 1 ♀; Upper Jurong Rd, NTI Campus; 18 Mar. 1981; Y.H. Koo leg.; ZRC_DER_0000084. • 1 ♂; Bukit Timah Forest; 3 Nov. 1974; D.H. Murphy leg.; Under bark; ZRC_DER_0000134. • 1 ♂; Bukit Timah Forest; 3 Dec. 1974; D.H. Murphy & L.Q.*R. leg*.; Under Dialiom log; ZRC_DER_0000135. • 1 ♂; same data as for ZRC_DER_0000134; ZRC_DER_0000136. • 1 ♀; same data as for ZRC_DER_0000134; ZRC_DER_0000137. • 1 ♀; same data as for ZRC_DER_0000134; ZRC_DER_0000138. • 1 nymph; same data as for ZRC_DER_0000134; ZRC_DER_0000139. • 1 nymph; same data as for ZRC_DER_0000134; ZRC_DER_0000140. • 1 ♀; same data as for ZRC_DER_0000134; ZRC_DER_0000141. • 1 ♂; Bukit Timah Forest; 17 May 1973; D.H. Murphy, B. W. Leonard leg.; Dead log; ZRC_DER_0000142. • 1 ♂; same data as for ZRC_DER_0000142; ZRC_DER_0000143. • 1 ♂; same data as for ZRC_DER_0000142; ZRC_DER_0000144. • 1 ♂; Bukit Timah Forest; 3 Dec. 1974; D.H. Murphy leg.; ZRC_DER_0000145. • 1 ♂; Windsor Nature Park; 7 Mar. 2024; Y. Kamimura leg.; Hand collection (day); ZRC_ENT00064390. • 1 ♀; same data as for ZRC_ENT00064390; ZRC_ENT00064391. • 1 ♀; same data as for ZRC_ENT00064390; ZRC_ENT00064392. • 1 ♀; same data as for ZRC_ENT00064390; ZRC_ENT00064393. • 1 ♂; Lower Peirce Reservoir Nature Park; 8 Mar. 2024; Y. Kamimura leg.; Hand collection (day); ZRC_ENT00064398. • 1 ♀; same data as for ZRC_ENT00064398; ZRC_ENT00064399. • 1 ♀; same data as for ZRC_ENT00064398; ZRC_ENT00064400. • 1 ♀; same data as for ZRC_ENT00064398; ZRC_ENT00064401. • 1 ♀; same data as for ZRC_ENT00064398; ZRC_ENT00064402. • 1 ♀; same data as for ZRC_ENT00064398; ZRC_ENT00064403. • 1 ♀; same data as for ZRC_ENT00064398; ZRC_ENT00064404. • 1 ♀; same data as for ZRC_ENT00064398; ZRC_ENT00064405. • 1 ♀; same data as for ZRC_ENT00064398; ZRC_ENT00064406. • 1 ♀; same data as for ZRC_ENT00064398; ZRC_ENT00064407. • 1 nymph; same data as for ZRC_ENT00064398; ZRC_ENT00064408. • 1 ♂; Thomson Nature Park; 8 Mar. 2024; Y. Kamimura leg.; Hand collection (day); ZRC_ENT00064416.

##### Distribution.

Philippines, Peninsular Malaysia, Borneo, Sumatra, Java, Sulawesi, New Guinea, Australia, Singapore.

### Subfamily Sparattinae Verhoeff, 1902a

**Tribe Auchenomini Burr, 1909**

***Auchenomus* Karsch, 1886**

#### Auchenomus setulosus

Taxon classificationAnimaliaDermapteraSpongiphoridae

(Burr, 1900)

933690FB-2F43-5F2D-BFCD-CCFA628D725B

Sparatta setulosa Burr, 1900a: 92 (Sarawak, Borneo).
Auchenomus setulosus : Burr 1911b: 59.
Auchenomus fulvus Borelli, 1915: 5 (Palawan, Philippines).

##### Literature.

Borelli (1921: 7): “1♂ da Singapore”; Borelli (1932b: 89): “Singapore, Avril 1923, (Saunders) l♂. Espèce déjà signalée de Singapore.”

##### Distribution.

Borneo, Philippine Islands (Palawan), Singapore, Sumatra (tentative identification based on a single female sample: Boeseman 1954).

#### Auchenomus

Taxon classificationAnimaliaDermapteraSpongiphoridae

sp.

8E9A1B20-6EC8-5673-B79C-08F7DD1162D1

##### Literature.

Haas et al. (2012: 96) reported wing unfolding behavior of possibly females of *A. setulosus* (as *Auchenomus* sp.), recorded near Venus Drive in Singapore.

### Tribe Chaetospaniini Steinmann, 1990

***Chaetospania* Karsch, 1886**

#### Chaetospania javana

Taxon classificationAnimaliaDermapteraSpongiphoridae

Borelli, 1926

5FC24EE9-5CB5-56CF-ABD0-1453FD46E9FF

Chaetospania javana Borelli, 1926c: 261 (Java).

##### Specimens examined.

**ZRC** • 2 ♂♂, 1 ♀; Bukit Timah Hindhede Drive; NA Dec. 1989; H.K. Lua leg.; ZRC_DER_0000002. • 2 ♂♂, 5 nymphs; Lower Peirce Reservoir; 21 Jul. 1990; C.M. Yang leg.; ZRC_DER_0000005. • 1 ♂; Nee Soon; 9 May 1992; ZRC_DER_0000080.

##### Distribution.

Peninsular Malaysia (Penang Island), Java, Singapore (new record).

##### Remarks.

The placement of *Chaetospania* within the tribe Chaetospaniini (subfamily Sparattinae) follows Steinmann (1990). The relatively large *Chaetospania* species examined in this study, characterized by a distinctly narrow pronotum relative to the tegmina, closely resembles *C. borneensis* (Dubrony, 1879). However, according to the original description (Dubrony 1879a), males of *C. borneensis* possess a single, conspicuous tooth located ~ 1/3 of the way from the base on each branch of the forceps, whereas in the holotype of *C. javana* (Sakai 1991), as well as in the Singaporean specimens examined herein, the tooth is situated approx. halfway from the base. Regarding male genitalia, only one illustration of *C. borneensis* is available in Steinmann (1990), depicting a characteristically elongate basal piece (referred to as the central parameral plate). In contrast, the genitalia of *C. javana* conform to the typical morphology of the genus (Kamimura et al. 2016a).

### Subfamily Labiinae Burr, 1909

***Spirolabia* Steinmann, 1987**

#### Spirolabia pilicornis

Taxon classificationAnimaliaDermapteraSpongiphoridae

(Motschulsky, 1863)

01A73652-E3C7-510C-9B45-F3A3665824DB

Labia pilicornis Motschulsky, 1863: 2 (Sri Lanka).
Labia rogenhoferi de Bormans, in Burr, 1903: 238 (Unknown locality, equatorial).
Labia minor (nec Linnaeus, 1758: 423): Rehn and Hebard 1914: 377 [misidentification] (Florida).
Labia rehni Hebard, 1917c: 317 (Florida).
Spirolabia pilicornis : Steinmann 1987: 182.
Circolabia pillicornis [sic.]: Srivastava, 1996: 102.

##### Specimens examined.

**ZRC** • 1 ♀; Kent Ridge; 11 Nov. 1991; Jean W.H. Yong leg.; ZRC_DER_0000056. • 1 ♂; Kent Ridge, NUS, Sheares Hall; 9 Dec. 1991; C.O. Hee leg.; ZRC_DER_0000061.

##### Distribution.

India, Sri Lanka, Philippines, Peninsular Malaysia, Borneo, Sumatra, Simalur Archipelago, Java, Sulawesi, Hawaii, Society Islands, Marquesas Islands, Guam, Mauritius, Singapore (new Record), Florida. Recently also recorded from southern Japan (Nishikawa 2025).

##### Remarks.

For the generic classification of Labiinae, we follow Steinmann (1990).

### *Paralabellula* Kevan, 1997 (= *Paralabella* Steinmann, 1990; see Kevan and Vickery 1997)

#### Paralabellula curvicauda

Taxon classificationAnimaliaAmoebidaSpongiphoridae

(Motschulsky, 1863)

64B955C0-D551-55D0-8850-C128245F2DE6

Forficula pygmaea Fabricius, 1793: 3 (Guinea).
Forfiscelia curvicauda Motschulsky, 1863: 2 (Nura-Ellia [= Nuwara Eliya, Sri Lanka]).
Forfiscelia dilaticauda Motschulsky, 1863: 3 (Nura-Ellia [= Nuwara Eliya, Sri Lanka]).
Platylabia guineensis Dohrn, 1867: 348 (insula Princiois [=Príncipe Island]).
Platylabia dimidiata Dohrn, 1867: 348 (Luzon, Philippines).
Labia curvicauda : de Bormans and Krauss 1900: 70.
Labia flavicollis de Bormans, in Burr, 1903: 235 (Samoa).
Platylabia camerunensis Borg, 1904: 570, pl. 26, fig. 4 (Cameroon).
Platylabia dimidiata var. camerunensis : Borelli, 1907: 382.
Platylabia dimidiata var. guineensis : Borelli 1907: 382.
Labia rechingeri Holdhaus, 1909: 541 (Samoa).
Labia curvicauda var. flavicollis : Boeseman 1954: 77.
Paralabella curvicauda : Steinmann 1990: 497.
Circolabia curvicauda : Srivastava, 1996: 102.
Paralabellula curvicauda : Kevan and Vickery 1997: 318.

##### Specimens examined.

**ZRC** • 1 ♀; Bukit Timah Hindhede Drive; NA Dec. 1989; H.K. Lua leg.; ZRC_DER_0000004. • 1 ♂; Bukit Timah Forest; 3 Nov. 1974; D.H. Murphy leg.; Under bark; ZRC_DER_0000108. • 1 ♀; Bukit Timah Forest; 3 Nov. 1974; D.H. Murphy leg.; Under bark; ZRC_DER_0000110. • 1 ♂; Bukit Timah Forest; 3 Dec. 1974; D.H. Murphy leg.; ZRC_DER_0000111. • 1 ♂; same data as for ZRC_DER_0000110; ZRC_DER_0000113. • 1 ♂; same data as for ZRC_DER_0000111; ZRC_DER_0000114. • 1 ♂; same data as for ZRC_DER_0000110; ZRC_DER_0000115. • 1 ♂ (brown morph); Thomson Nature Park; 8 Mar. 2024; Y. Kamimura leg.; Hand collection (day); ZRC_ENT00064424. • 1 ♂ (black morph); same data as for ZRC_ENT00064424; ZRC_ENT00064425. • 1 ♂ (black morph); Thomson Nature Park; 12 Mar. 2024; Y. Kamimura leg.; Hand collection (day); ZRC_ENT00064440. • 1 ♀ (black morph); same data as for ZRC_ENT00064440; ZRC_ENT00064441. • 1 ♀; same data as for ZRC_ENT00064440; ZRC_ENT00064442. **NTU** • 1 ♀; Central catchment nature reserve; 14 Apr. 2023; W.N. Lam, G.K. Png, J. L. Loo leg.; Hand collected, from deadwood; MAC6F-HC_23_1. • 1 ♂; same data as for MAC6F-HC_23_1; MAC6F-HC_23_2. • 1 ♀; same data as for MAC6F-HC_23_1; MAC6F-HC_23_3. • 1 ♀; same data as for MAC6F-HC_23_1; MAC6F-HC_23_4.

##### Distribution.

Circum-tropical cosmopolitan. New record to Singapore.

##### Remarks.

Steinmann (1990) established the genus *Paralabella* for labiine earwigs characterized by setulose branches of the forceps, a more or less straight virga, and parameres that are not apically excised. The designated type species was *Forficula annulata* Fabricius, 1793. However, this species is evidently an anisolabidid, and is herein treated under the genus *Euborellia* (Brindle 1981; Kevan and Vickery 1997). Consequently, *Paralabella* Steinmann, 1990 is considered a junior synonym of *Euborellia*. To accommodate the remaining species formerly placed in *Paralabella* (excluding *P. annulata*), Kevan (in Kevan and Vickery 1997) proposed the genus *Paralabellula*, designating a new type species.

At least two distinct color morphs of *Paralabellula curvicauda* are recognized, one with a blackish pronotum and the other with a yellow-brownish pronotum, accompanied by differences in male genital morphology (Srivastava 2013). These morphs may represent separate species. Both forms occur in Singapore (blackish morph: ZRC_ENT00064440–ZRC_ENT00064442, ZRC_ENT00064425; brownish morph: ZRC_ENT00064424), although specimens exhibiting advanced discoloration due to age were not conclusively identified.

### *Paraspania* Steinmann, 1985

#### Paraspania emarginata

Taxon classificationAnimaliaDermapteraSpongiphoridae

(Srivastava, 1978)

EE205C85-7C41-544D-9543-70FFAACB7781

Figs 35–38

Labia emarginata Srivastava, 1978: 278, figs 32–35 (Culion, Philippines).
Paraspania emarginata : Steinmann 1990: 519.
Circolabia emarginnta [sic.]: Srivastava 1996: 102.

##### Specimens examined.

**ZRC** • 1 ♂; Thomson Nature Park; 12 Mar. 2024; Y. Kamimura leg.; Hand collection (day); ZRC_ENT00064427.

##### Distribution.

Philippines (Culion, Mindoro), Singapore (new record).

##### Remarks.

Steinmann (1985) established the genus *Paraspania* to accommodate labiine species characterized by a spirally coiled virga, branches of the forceps that are not strongly setulose, and the absence of a longitudinal protuberance on the penultimate sternite. He transferred several species previously placed in *Chaetospania* and *Sparatta* Audinet-Serville, 1839 (Sparattinae), together with *Labia emarginata* Srivastava, 1978 (Steinmann 1990). Srivastava (1996), however, treated *Paraspania* as a synonym of *Chaetospania*, placing it within Labiinae, contrary to Steinmann’s (1990) classification, which assigned *Chaetospania* and *Paraspania* to Sparattinae and Labiinae, respectively. This taxonomic inconsistency stems from different interpretations of morphological traits, particularly body shape and virgal structure, in genus-level classification.

Species assigned to *Chaetospania* and *Paraspania*, especially the latter, generally lack the pronounced dorsoventral flattening seen in members of Auchenomini and Sparattini. Moreover, the degree of virga coiling and the extent of forceps setation, used by Steinmann (1985) to distinguish *Chaetospania* from *Paraspania*, and by Srivastava (2013) to separate *Chaetospania* from *Circolabia*, exhibit continuous variation across species, complicating the delineation of generic boundaries. Resolving these issues will require further investigation, particularly through molecular phylogenetic analyses. For the purposes of this study, and to facilitate species identification while avoiding overly broad generic definitions, we tentatively follow Steinmann’s classification.

**Figures 35–38.**
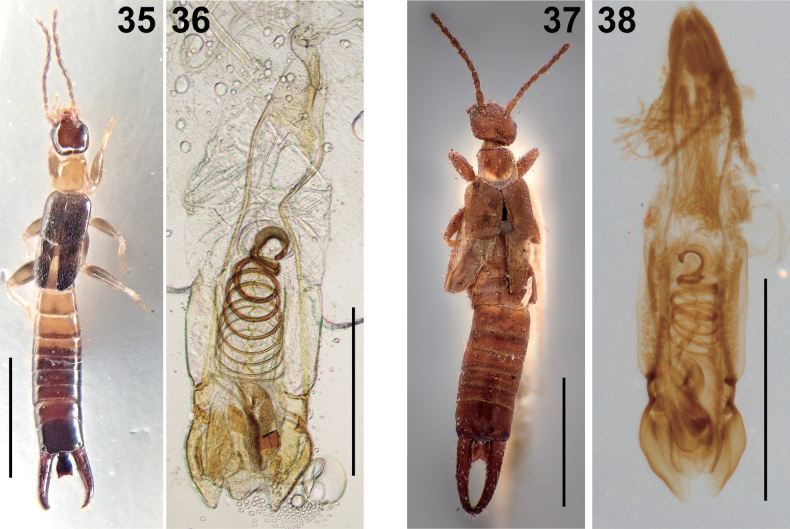
*Paraspania emarginata* (Srivastava, 1978). **35, 36**. Male collected in Singapore (ZRC_ENT00064427): **35**. Dorsal habitus; **36**. Genitalia; **37, 38**. Holotype (male) of *Labia emarginata* Srivastava, 1978 (FMNH-INS 0000095742): **37**. Dorsal habitus **38**. Genitalia. Scale bars: 2 mm (**35, 37**); 0.5 mm (**36, 38**).

A single male specimen of *Paraspania* collected in Singapore (ZRC_ENT00064427; Figs 35, 36) shows no discernible morphological differences from the holotype of *Paraspania emarginata* (= *Labia emarginata*), previously known only from Culion and Mindoro Islands in the Philippines (FMNH-INS 0000095742 [FMNH]: Figs 37, 38; Srivastava 1978, 1990). Despite the considerable geographic separation (over 2,000 km), we tentatively identify the Singapore specimen as *P. emarginata*.

### *Sphingolabis* de Bormans, 1883

#### Sphingolabis hawaiiensis

Taxon classificationAnimaliaDermapteraSpongiphoridae

(de Bormans, 1882)

B1F73CDC-CF2E-536E-959E-6120482A0823

Forficula hawaiensis de Bormans, 1882: 341, figs (Hawaii).
Apterygida hawaiensis : de Bormans and Krauss 1900: 114.
Sphingolabis hawaiiensis : Burr 1911b: 55.

##### Literature.

Burr (1902: 486) as *Apterygida hawaiiensis*: “*A. hawaiiensis* (Borm.). – Singapore (Biró).”

##### Distribution.

Philippine Islands to New Guinea (Solomon Islands), Lombok, Hawaii Islands, Sandwich Islands, New Hebrides.

### Subfamily Spongiphorinae Verhoeff, 1902a

***Spongovostox* Burr, 1911b**

#### Spongovostox semiflavus

Taxon classificationAnimaliaDermapteraSpongiphoridae

(de Bormans, 1894)

8690084E-FE67-539D-B311-F636FAE33F89

Spongophora semi-flava de Bormans, 1894: 385 (Myanmar).
Spongiphora semiflava : de Bormans and Krauss 1900: 59.
Spongovostox semiflavus : Burr 1911c: 783.
Spongovostox semiflavus var. dentifera : Borelli 1926b: 66.
Spongovostox semiflavus var. crenata : Borelli 1926b: 66.
Apovostox semiflavus : Hebard 1927: 32.
Apovostox semiflavos [misprint]: Sakai 1970b: 54.
Spongovostox semiflavos [misprint]: Srivastava 1976: 31.

##### Specimens examined.

**ZRC** • 1 ♂; Thomson Nature Park; 8 Mar. 2024; Y. Kamimura leg.; Hand collection (day); ZRC_ENT00064417. • 1 ♂; same data as for ZRC_ENT00064417; ZRC_ENT00064418. • 1 ♀; same data as for ZRC_ENT00064417; ZRC_ENT00064419. • 1 ♀; same data as for ZRC_ENT00064417; ZRC_ENT00064420. • 1 ♀; same data as for ZRC_ENT00064417; ZRC_ENT00064421. • 1 ♀; same data as for ZRC_ENT00064417; ZRC_ENT00064422. • 1 ♂; same collection data as for ZRC_ENT00064417; 12 Mar. 2024; ZRC_ENT00064429. • 1 ♀; same data as for ZRC_ENT00064429; ZRC_ENT00064430. • 1 ♀; same data as for ZRC_ENT00064429; ZRC_ENT00064431. • 1 ♀; same data as for ZRC_ENT00064429; ZRC_ENT00064432. • 1 ♀; same data as for ZRC_ENT00064429; ZRC_ENT00064433.

##### Distribution.

India, Sri Lanka, Bhutan, Myanmar, China (Yunnan), Laos, Thailand, Vietnam, Peninsular Malaysia, Borneo, Sumatra, Java, Simalur, Sumba, Philippines (Palawan, Mindanao, Luzon), Taiwan, Bismarck Island, Singapore (new record).

#### Spongovostox

Taxon classificationAnimaliaDermapteraSpongiphoridae

sp.

03B44C23-5891-522F-BAFC-E4692656CA24

##### Specimens examined.

**ZRC** • 1 nymph; Thomson Nature Park; 8 Mar. 2024; Y. Kamimura leg.; Hand collection (day); ZRC_ENT00064423.

##### Remarks.

The specimens are likely nymphs of *S. semiflavus*. However, because multiple species of this genus may occur within a single decayed log, a more detailed examination is required for species-level identification.

### *Marava* Burr, 1911a

#### Marava arachidis

Taxon classificationAnimaliaDermapteraSpongiphoridae

(Yersin, 1860)

7FC1F75C-D7D2-5C20-A2E4-DBDD19DB1A34

Forficula arachidis Yersin, 1860: 509, pl. 10 figs 33–35 (France).
Forficula nigripennis Motschulsky, 1863: 1 (Montagnes de Nura-Ellia, Ceylan [= Sri Lanka]).
Forficula wallacei Dohrn, 1865: 88 (New Guinea).
Forficula (Apterygida) gravidula Gerstaecker, 1873: 50, pl. 3 fig. 9 (Mombas, Ost-Africa [= Mombasa, East Africa (Kenya)]).
Labia brunnea Scudder, 1876b: 264 (Cuba).
Labia grandis Dubrony, 1879a: 366, figs (Somerset, Australia).
Chelidula arachidis : Finot 1890: 70.
Sphingolabis arachidis : de Bormans 1894: 406.
Apterygida arachidis : de Bormans & Krauss, 1900: 117.
Prolabia arachidis : Burr 1911a: 60.
Marava grandis : Burr 1911a: 60.
Prolabia ascensionis Hebard, 1917a: 243, pl. 16, figs 8, 9 (Ascension Island, South Atlantic).
Marava arachidis : Hincks, 1954: 162.
Marava ascensionis : Sakai 1970b: 173.
Marava wallacei : Steinmann 1983: 49.

##### Specimens examined.

**ZRC** • 1 ♂; Linden Drive Kampong; 9 Mar. 1975; D.H. Murphy leg.; ZRC_DER_0000159.

##### Distribution.

Almost all faunal regions. New record to Singapore.

### Family Chelisochidae Verhoeff, 1902a

**Subfamily Chelisochinae Verhoeff, 1902a**

***Chelisoches* Scudder, 1876a**

#### Chelisoches morio

Taxon classificationAnimaliaDermapteraChelisochidae

(Fabricius, 1775)

A185C667-CA7A-5E28-8B3D-5DA350FA047C

Forficula morio Fabricius, 1775: 270 (Tahiti).
Lobophora rufitarsis Audinet-Serville, 1839: 33 (Java).
Forficula (Psalidophora) albomarginata de Haan, 1842: 241 (Batang Singalang [Singgalang, West Sumatra]).
Forficula tasmanica Blanchard, 1853: 350, pl. 1 fig. 2 (Tasmania).
Lobophora nigro-nitens Stål, 1860: 305 (Java).
Lobophora cincticornis Stål, 1860: 305 (Mauritius).
Lobophora tartarea Stål, 1860: 305 (Taiti [=Tahiti]).
Lobophora morio : Dohrn, 1865: 71.
Chelisoches comprimens Scudder, 1876a: 252 (Africa).
Labidura nigricornis Kirby, 1888: 546 (Christmas Island).
Chelisoches morio : de Bormans 1888: 440.
Sphingolabis insularis Kirby, 1900 in Distant (1900): 13, pl. 1 fig. 3 (Pemba Island).
Chelisoches rufitarsis : Kirby 1904: 33.
Chelisoches cincticollis : Kirby 1904: 33.
Chelisoches albomarginatus : Kirby 1904: 34.
Chelisoches nigricornis : Kirby 1904: 34.
Chelisoches tasmanicus : Kirby 1904: 35.
Chelisoches stratioticus Rehn, 1906: 509, fig. 6 (Trong, Lower Siam [= northern part of the Malay Peninsula]).
Chelisoches lilyanus Holdhaus, 1909: 541 (Tenasserim, Birma [= Myanmar]).
Chelisoches tigris Burr, 1913a: 143 (Rotung, Abor [= Arunachal Pradesh, India]).
Chelisoches imitator Ramamurthi, 1967: 250 (Lavongai, Bismarck Islands [= Lavongai, Papua New Guinea]).

##### Literature.

Kirby (1904: 33) as *Chelisoches rufitarsis*.

##### Specimens examined.

**ZRC** • 1 nymph; Bukit Batok, House, GP; 15 Aug. 2000; NUS DBS students leg.; In house; ZRC_DER_0000058. • 1 ♂; Bukit Timah Hindhede Drive; 19 Dec. 1988; H.K. Lua leg.; ZRC_DER_0000076. • 1 ♂; 1980; ZRC_DER_0000093. • 1 nymph; Telok Blangah; 7 Jan. 1977; K.*T. leg*.; House wall; ZRC_DER_0000118. • 1 nymph; University campus; 13 Jun. 1976; D.H. Murphy leg.; On male flowers of oil palm; ZRC_DER_0000119. • 1 ♀; University campus; 15 Jun. 1976; D.H. Murphy leg.; On flower of *Baccaurea*; ZRC_DER_0000120.

##### Distribution.

Circum-tropical cosmopolitan.

### *Proreus* Burr, 1907

#### Proreus simulans

Taxon classificationAnimaliaDermapteraChelisochidae

(Stål, 1860)

82E0415B-68EB-52A8-82E9-2D80EB4E10A2

Forficula simulans Stål, 1860: 302 (Java).
Forficula modesta Stål, 1860: 302 (Hong Kong).
Lobophora simulans : Dohrn 1865: 74.
Lobophora modesta : Dohrn 1865: 74.
Chelisoches simulans : Scudder 1876c: 309.
Spongiphora sphinx Burr, 1900a: 91 (Kuching, Sarawak [= Borneo]).
Spongiphora sphinx : Kirby 1904: 30.
Labidurodes formosanus Shiraki, 1906: 92, fig. 2 (Formosa [= Taiwan]).
Labidurodes okinawaiensis Shiraki, 1907: 189 (Okinawa, Japan).
Erotesis sphinx : Burr 1910b: 114.
Proreus simulans : Burr 1910b: 137.
Proreus simulans var. modestus : Borelli 1926c: 267.

##### Specimens examined.

**ZRC** • 1 ♀; 1992; ZRC_DER_0000029. • 1 ♀; Ulu Sembawang; 23 Jan. 1976; D. Bina leg.; Sugar cane; ZRC_DER_0000103. • 1 ♀; same data as for ZRC_DER_0000103; ZRC_DER_0000104. • 1 ♂; Bukit Panjang; 19 Jan. 1976; D.H. Murphy leg.; Sugar cane; ZRC_DER_0000105. • 1 ♂; Bukit Panjang; 11 Jan. 1977; H.M.W. leg.; Sugar cane plantation; ZRC_DER_0000112.

##### Distribution.

Widely distributed in Indomalayan region. New record to Singapore.

#### Proreus ludekingi

Taxon classificationAnimaliaDermapteraChelisochidae

(Dohrn, 1865)

78B560FC-5278-5D91-9BDA-6318E91FC43E

Lobophora ludekingi Dohrn, 1865: 73 (Sumatra).
Chelisoches ludekingi : de Bormans 1884b: 199.
Proreus ludekingi : Burr 1907: 131.

##### Literature.

Borelli (1932b: 90): “Aug. 1922, ♂ de la variété microlabia Bormans.”

##### Distribution.

Philippines, Peninsular Malaysia, Borneo, Sumatra, Java, Singapore.

### *Hamaxas* Burr, 1907

#### Hamaxas feae

Taxon classificationAnimaliaDermapteraChelisochidae

(de Bormans, 1894)

ABC42D8D-7E36-519E-992D-421CA3DA81DA

Chelisoches feae de Bormans, 1894: 393 (Carin Chebà [= Myanmar]).
Hamaxas feae : Burr 1907: 134.

##### Specimens examined.

**ZRC** • 1 ♀; Bukit Timah Forest, JFV streamside; 18 Jul. 1979; D.H. Murphy leg.; ZRC_DER_0000130. • 1 ♀; Sime road, KPG; 15 Oct. 1974; D.H. Murphy leg.; ZRC_DER_0000131. • 1 ♀; Bukit Timah Forest; 5 Aug. 1974; D.H. Murphy leg.; ZRC_DER_0000132. **NTU** • 1 ♀; Central catchment nature reserve; 2023; S. Yap leg.; Flight intercept trap; FIT173_23_1.

##### Distribution.

India, Myanmar, China (Yunnan), Vietnam, Thailand, Philippines, Borneo, Sumatra, Java, New Guinea, New Zealand, Singapore (new record).

### Family Forficulidae Latreille, 1810

**Subfamily Opisthocosmiinae Verhoeff, 1902a**

***Hypurgus* Burr, 1907**

#### Hypurgus humeralis

Taxon classificationAnimaliaDermapteraForficulidae

(Kirby, 1891)

530A913F-7743-51A6-BE6F-CEA25FAD2E87

Opisthocosmia humeralis Kirby, 1891: 523 (Ceylon [= Sri Lanka])
Hypurgus humeralis : Burr 1907: 101.
Hypurgus humeralis var. vittatus : Burr 1911c: 799.
Sadiya grata Hebard, 1923: 232, pl. 21 figs 25, 26 (Sadiya, Assam, India).
Hypurgus grata : Srivastava 1976: 69.

##### Specimens examined.

**ZRC** • 1 ♀; Yishun Ave 2; 7 Nov. 2012; K.R. Tan leg.; Forest patch, on tall grass; ZRC_DER_0000066. • 1 ♀; Nee Soon Swamp Forest; 28 May 1992; UV light trap; ZRC_DER_0000079. **NTU** • 1 ♀; Central catchment nature reserve; 16 May 2023; W.N. Lam, G.K. Png, J. L. Loo leg.; Winkler extraction of leaf litter; PEI2F_23_1. • 1 ♀; Pulau Ubin; 12 May 2023; W.N. Lam, G.K. Png, J. L. Loo leg.; Winkler extraction of leaf litter; UBI3U_23_1.

##### Distribution.

India, Nepal, Sri Lanka, Myanmar, China (Yunnan), Thailand, Vietnam, Peninsular Malaysia, Borneo, Singapore (new Record).

### *Timomenus* Burr, 1907

#### Timomenus bicuspis

Taxon classificationAnimaliaDermapteraForficulidae

(Stål, 1860)

1A964D4D-5AB1-551B-9EE6-A602328C9FE5

Forficula bicuspis Stål, 1860: 301 (Java).
Apterygida bicuspis : de Bormans and Krauss 1900: 114.
Sphingolabis bicuspis : Kirby 1904: 46.
Timomenus bicuspis : Burr 1907: 96.

##### Specimens examined.

**ZRC** • 1 ♂; Dairy Farm Rd. spring feed; 15 Oct. 1996; C.M. Yang leg.; ZRC_DER_0000026.

##### Distribution.

Peninsular Malaysia, Java, Singapore (new record).

### *Pareparchus* Burr, 1911b

#### Pareparchus minusculus

Taxon classificationAnimaliaDermapteraForficulidae

(de Bormans, 1884)

976F9ABF-E80F-518A-AF27-D8BDDC56865C

Opisthocosmia minuscula de Bormans, 1884a: 190 (Agam [= Agam, West Sumatra]).
Eparchus minuscula : Burr, 1907: 121.
Pareparchus minusculus : Burr 1911b: 92.

##### Literature.

Burr (1902: 485) as *Opisthocosmia minuscula*: “*O. minuscula* Dohrn. – Singapore (Xántus).”

##### Distribution.

Sumatra, Borneo, Singapore.

### Order ZORAPTERA Silvestri, 1913

**Family Spiralizoridae Kočárek, Horká & Kundrata, 2020**

**Subfamily Spiralizorinae Kočárek, Horká & Kundrata, 2020**

***Spiralizoros* Kočárek, Horká & Kundrata, 2020**

#### Spiralizoros caudelli

Taxon classificationAnimaliaZorapteraSpiralizoridae

(Karny, 1927)

6FD15F2F-C54C-54A5-B5E2-5A57BC69E0FE

“*Zorotypus* aus Wai Lima” Karny, 1922: 15.
Zorotypus caudelli Karny, 1927: 1, pl. 1 (Sumatra).
Spiralizoros caudelli : Kočárek et al. 2020: 13.

##### Specimens examined.

**ZRC** • 1 ♂; Thomson Nature Park; 8 Mar. 2024; Y. Kamimura leg.; Hand collection (day); ZRC_ENT00064443. • 1 ♂; same data as for ZRC_ENT00064443; ZRC_ENT00064444. • 1 ♂; same data as for ZRC_ENT00064443; ZRC_ENT00064445. • 1 ♀; same data as for ZRC_ENT00064443; ZRC_ENT00064446. • 1 ♀; same data as for ZRC_ENT00064443; ZRC_ENT00064447. • 1 ♀; same data as for ZRC_ENT00064443; ZRC_ENT00064448. • 1 ♀; same data as for ZRC_ENT00064443; ZRC_ENT00064449. • 1 ♀; same data as for ZRC_ENT00064443; ZRC_ENT00064450.

##### Distribution.

Sumatra, Borneo, Peninsular Malaysia, Singapore (new record). However, see Kočárek et al. (2020: 14) for a discussion of the taxonomic ambiguities in this group.

#### Zoraptera

Taxon classificationAnimaliaZorapteraSpiralizoridae

sp.

6A2DFDBF-33EE-5355-9575-44162D76A048

##### Specimens examined.

**ZRC** • 5 individuals [sex-stage unspecified]; Bukit Timah Nature Reserve, Cave Path; 25 May 2022; C. Lucanas leg.; Hand collection (day); ZRC_ENT00015570.

##### Remarks.

It is likely that these specimens belong to *S. caudelli*, but a detailed examination of genital morphology and other characteristics is necessary for a more precise identification.

## Discussion

Among the species listed above, *N. ridleyi* (as *Diplatys ridleyi*), *P. major*, *G. electa*, *N. amoenus*, *M. arachidis*, *C. morio*, *H. feae*, and *P. simulans* were reported by Wang (2011) as occurring in Singapore, although no detailed collection or observation records were provided. *Auchenomus* sp. was also included in Wang’s list, but it remains unclear whether it corresponds to the same taxon recorded in our study. Additionally, Wang listed several species not included in our records, namely, “*Euborellia annulipes*,” “*Labia* sp.,” “*Chelisoches momo*,” and “striped earwig of Labiduridae.” The latter is presumed to refer to *Labidura riparia*. Although *Euborellia annulipes* (Lucas, 1847) is a cosmopolitan species and may plausibly occur in Singapore, we have not confirmed any reliable specimens. Furthermore, we were unable to locate a formal description of a species named *Chelisoches momo*. Given these uncertainties, we report in this work only those species for which reliable specimen records are available.

Our examination revealed two notable characteristics of the dermapteran fauna of Singapore. First, as expected given the country’s geographic location, many species are shared with Peninsular Malaysia. Of the 29 dermapteran species recorded from Singapore and identified to the species level, 22 (75.9%) are also known from Peninsular Malaysia. Among the remaining seven species, *N. ridleyi* is endemic to Singapore and *P. emarginata* has been known only from Philippines. The other five species (*E. peterseni*, *G. minor*, *A. setulosus*, *S. hawaiiensis*, and *P. minusculus*) have also been recorded from Java, Sumatra, and/or Borneo. The apparent uniqueness of Singapore’s fauna may be partly attributable to insufficient survey efforts in southern Peninsular Malaysia. Continued research in regions such as Johor state and within Singapore is therefore recommended.

Another notable feature of Singapore’s dermapteran fauna is the high proportion of species with broad distributions across Southeast Asia, East Asia, or even wider regions. This pattern is also evident in Zoraptera: *Spiralizoros caudelli* is one of the commonest zorapteran species in Peninsular Malaysia (Mashimo et al. 2013). Such widespread distributions are presumably linked to urbanization in Singapore, which has led to the fragmentation and isolation of natural forest habitats.

Penang Island (Peninsular Malaysia; 299 km^2^), representing ~ 41% of Singapore’s land area, has recorded 31 species of Dermaptera identified to the species level (Kamimura et al. 2016a, b). Despite differences in survey effort, Singapore’s species count (29 species identified to the species level) is comparatively lower given its larger area. Notably, surveys on Penang Island have reported the highest species diversity in secondary forest environments, including fruit plantations (Kamimura et al. 2016a), whereas such habitats are currently scarce in Singapore (Corlett 1992). This habitat limitation may further contribute to the reduced diversity of Dermaptera, and potentially Zoraptera, in Singapore.

Historically, two species, *Nannopygia ridleyi* (= *Diplatys ridleyi*) and *Gonolabis emarginata*, were considered potentially endemic to Singapore. However, we propose that *G. emarginata* is a junior synonym of *G. sumatrana*, a species more broadly distributed across Southeast Asia. Importantly, this reassessment does not preclude the existence of yet-undiscovered endemic species of Dermaptera or Zoraptera in Singapore. An intensive field survey that we conducted in 2024 led to the discovery of five species (*E. roseiventre*, *P. infernalis*, *A. chartaceus*, *S. semiflavus*, and *S. emarginata*) that had not previously been represented in museum collections. Arboreal species, in particular, appear to have been under-sampled, and further targeted research is strongly encouraged.

## Supplementary Material

XML Treatment for Nannopygia ridleyi

XML Treatment for Cranopygia

XML Treatment for Echinosoma roseiventre

XML Treatment for Parapsalis infernalis

XML Treatment for Apachyus chartaceus

XML Treatment for Apachyus

XML Treatment for Platylabia major

XML Treatment for Epilandex peterseni

XML Treatment for Euborellia annulata

XML Treatment for Gonolabis electa

XML Treatment for Gonolabis minor

XML Treatment for Gonolabis sumatrana

XML Treatment for Anisolabidinae

XML Treatment for Anisolabidinae

XML Treatment for Metisolabis punctata

XML Treatment for Labidura riparia

XML Treatment for Nala lividipes

XML Treatment for Nesogaster amoenus

XML Treatment for Auchenomus setulosus

XML Treatment for Auchenomus

XML Treatment for Chaetospania javana

XML Treatment for Spirolabia pilicornis

XML Treatment for Paralabellula curvicauda

XML Treatment for Paraspania emarginata

XML Treatment for Sphingolabis hawaiiensis

XML Treatment for Spongovostox semiflavus

XML Treatment for Spongovostox

XML Treatment for Marava arachidis

XML Treatment for Chelisoches morio

XML Treatment for Proreus simulans

XML Treatment for Proreus ludekingi

XML Treatment for Hamaxas feae

XML Treatment for Hypurgus humeralis

XML Treatment for Timomenus bicuspis

XML Treatment for Pareparchus minusculus

XML Treatment for Spiralizoros caudelli

XML Treatment for Zoraptera

## Additional information

### Use of AI

No use of AI was reported.

### Author ORCIDs

Yoshitaka Kamimura https://orcid.org/0000-0003-2882-491X

Shen Cheah https://orcid.org/0009-0007-7899-0306

Wendy Yanling Wang https://orcid.org/0000-0003-0745-4702

Weng Ngai Lam https://orcid.org/0000-0002-1188-1363

### Data availability

All of the data that support the findings of this study are available in the main text.
